# Characterization of Norovirus RNA replicase for *in vitro* amplification of RNA

**DOI:** 10.1186/1472-6750-13-85

**Published:** 2013-10-09

**Authors:** Hidenao Arai, Koichi Nishigaki, Naoto Nemoto, Miho Suzuki, Yuzuru Husimi

**Affiliations:** 1 Graduate School of Science and Engineering, Saitama University, 255 Shimo-Okubo, Sakura-ku, Saitama 338-8570, Japan; 2 Innovative Research Organization, Saitama University, 255 Shimo-Okubo, Sakura-ku, Saitama 338-8570, Japan

**Keywords:** RNA-dependent RNA polymerase, RNA replication, Isothermal RNA amplification, *in vitro* evolution

## Abstract

**Background:**

The isothermal amplification of RNA *in vitro* has been used for the study of *in vitro* evolution of RNA. Although Qβ replicase has been traditionally used as an enzyme for this purpose, we planned to use norovirus replicase (NV3D^pol^) due to its structural simplicity in the scope of *in vitro* autonomous evolution of the protein. Characteristics of the enzyme NV3D^pol^*in vitro* were re-evaluated in this context.

**Results:**

NV3D^pol^, synthesized by using a cell-free translation system, represented the activities which were reported in the previous several studies and the reports were not fully consistent each other. The efficiency of the initiation of replication was dependent on the 3’-terminal structure of single-stranded RNA template, and especially, NV3D^pol^ preferred a self-priming small stem-loop. In the non-self-priming and primer-independent replication reaction, the presence of -CCC residues at the 3’-terminus increased the initiation efficiency and we demonstrated the one-pot isothermal RNA (even dsRNA) amplification by 16-fold. NV3D^pol^ also showed a weak activity of elongation-reaction from a long primer. Based on these results, we present a scheme of the primer-independent isothermal amplification of RNA with NV3D^pol^*in vitro*.

**Conclusions:**

NV3D^pol^ can be used as an RNA replicase in *in vitro* RNA + protein evolution with the RNA of special terminal sequences.

## Background

*In vitro* evolution of RNA has been performed with a given replicase [1-3] or with a given RNA polymerase and a given reverse transcriptase [4-6]. *In vitro* evolution of RNA having a gene region encoding the replicase, that is, *in vitro* evolution of RNA with an evolvable replicase [7] is a next step approaching to synthetic biology on an origin of life. It may be one of the most fundamental experiments of *in vitro* evolution of a protein. *In vitro* evolution of a protein has to be coupled with *in vitro* evolution of its gene and usually is performed with a given translation system. If a synthetic biologist wishes to reconstruct the phenomenon of the origin of life, he should use primitive translation system which can evolve to high efficient system, but such a translation system is not available.

Coupling a protein evolution and its gene evolution is realized by a phenotype-genotype linking strategy in the selection process. There are four types of the strategy; ribozyme-type, virus-type, cell-type and external intelligence-type. The ribozyme-type is the strategy of phenotype-genotype linking by carrying both on an RNA [8,9]. The virus-type is the strategy by binding a protein to its gene [10,11]. The cell-type is the strategy by enclosing a protein and its gene in a compartment [7]. External intelligence-type is the strategy by measuring and picking the fittest protein [12].

*In vitro* evolution experiments can be classified into two categories; artificial selection-type and natural selection-type. The former is called directed evolution, in which the fitness measure is set by the experimenter. The fitness of the latter experiment is the specific growth rate. PCR is most convenient amplification method in the former experiments, but cannot be used in the latter experiments. One-pot isothermal amplification method is convenient in the latter, because a flow reactor experiment is possible in principle and a serial transfer experiment is easily performed as an approximation of the flow reactor experiment. Examples of the one-pot isothermal amplification are Qβ replicase method [1], 3SR [4], NASBA [5], RNA-Z [6], etc.

Qβ RNA replicase is from an RNA phage Qβ of *E.coli* and is a hetero-tetramer, in which only β subunit is encoded in the viral genome and other three subunits (EF-Tu, EF-Ts and S1) are recruited from the host-cell translation machinery [13]. For the effective replication of viral genome RNA, it requires a host factor Hfq, which binds to the specific site of the RNA and recruits the replicase [14]. When the isothermal amplification of the genomic RNA was performed in a test tube, a parasitic short RNA called RQ RNA emerged and it made the amplification of genomic RNA difficult [15]. Yomo and the co-workers demonstrated that the compartmentalization of the reaction solution into a water-in-oil emulsion inhibits the growth of the parasite [16].

Yomo’s compartment method is just a cell-type strategy of phenotype-genotype linking and was applied to an experiment of the coupled RNA/replicase evolution [16]. We are planning an experiment of the coupled RNA/replicase evolution by using an isothermal RNA amplification and a virus-type strategy called *in vitro* virus method or mRNA display [10,11]. This “*in vitro* virus” will evolve autonomously in a flow reactor. Qβ RNA replicase is not adequate for our purpose because it must recruit three proteins from the cell-free replication system and this situation makes population growth dynamics very complex in the test tube containing the cell free translation system. Norovirus RNA replicase (RNA-dependent RNA polymerase; NV3D^pol^) is a candidate for our *in vitro* virus, because it consists of a single polypeptide chain and its molecular weight is not so large (56 kDa).

There are several preceding reports on the characteristics of various modes of RNA replication catalyzed by the NV3D^pol^*in vitro*[17-20]. They reported on the activity of making of double-strand RNA through complementary strand polymerization on a single strand template with and without a primer, the activity of strand dissociation replication, the activity of making of a hairpin through self-priming and also the terminal nucleotidyl transferase (TNT) activity. Their reports were not consistent with each other.

Therefore we re-examined the activities of NV3D^pol^*in vitro* in order to investigate whether we can use this enzyme as an RNA replicase in evolution experiments *in vitro*, instead of Qβ replicase.

## Results

### Preparation of NV3D^pol^

**Figure 1:**
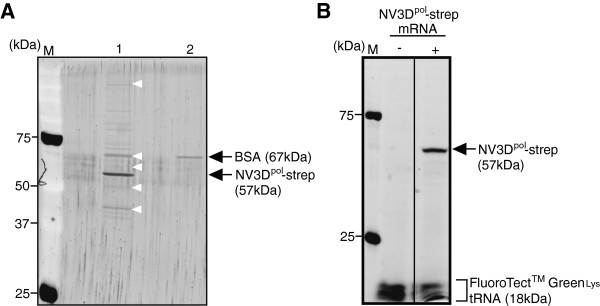
**Preparation of NV3D**^**pol**^**. (A)** SDS-PAGE analysis of NV3D^pol^-strep synthesized in the cell-free translation system and purified with Strep-Tactin Superflow Plus purification system (Lane1), and BSA (Lane2). M; protein molecular marker (Precision Plus Protein™ Dual Color Standards, Bio-Rad). SDS-PAGE was detected with SYPROruby (Invitrogen) staining. White arrowheads indicate the proteins from BSA solution (TaKaRa) used as carrier protein. **(B)** Same as **(A)** except incorporation of FluoroTect™ Green_Lys_ tRNA into NV3D^pol^-strep during the cell-free translation reaction and detected under a fluorescence detection mode. Translation reaction was run with or without the NV3D^pol^-strep mRNA.

The gene of our NV3D^pol^ was a gift from BML Inc., that is, it was same as Ref. [19]. After construction of pTD-NV3D^pol^-strep, we sequenced it and confirmed ours was triple mutant at amino acid sequence level from the sequence in Ref. [19], which was different from the sequence in Ref. [16] (See Additional file 1: Figure S1). As shown in Figure 1A, a band corresponding to about 57 kDa of NV3D^pol^-strep protein was observed on SDS-PAGE. From the incorporation of FluoroTect™ Green_Lys_ tRNA into the protein during the cell-free translation, only a sharp single band was detected under a fluorescence detection mode (Figure 1B). Therefore, NV3D^pol^-strep protein was synthesized correctly in the insect cell-free translation system. NV3D^pol^-strep protein modiied with Strep-tag II [21] at the C-terminus was able to be highly purified with Strep-tactin affinity chromatography (Figure 1A). In Figure 1A lane1, white arrowhead indicates the protein bands taken from BSA solution (TaKaRa). As shown in Additional file 2: Figure S2, NV3D^pol^ had an RNA-dependent RNA synthesis activity under the excess BSA condition. Therefore, NV3D^pol^ prepared here was not affected with contaminated proteins.

### RNA amplification reaction with NV3D^pol^

**Figure 2:**
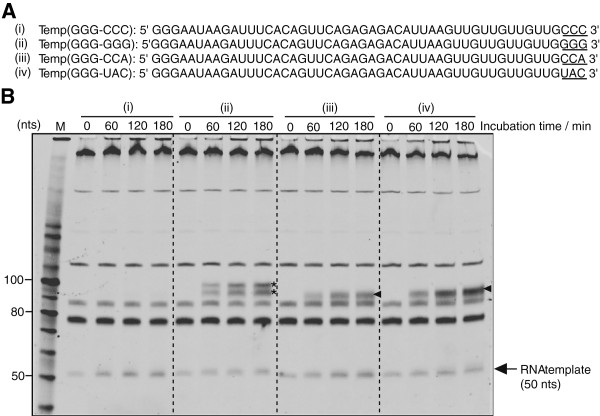
**Primer-independent RNA synthesis on RNA templates with NV3D**^**pol**^**. (A)** The sequence of RNA templates. **(B)** NV3D^pol^ (5 pmol) was incubated with **(i)** Temp(GGG-CCC), **(ii)** Temp(GGG-GGG), **(iii)** Temp(GGG-CCA) or **(iv)** Temp(GGG-UAC) (5 pmol each), and sampled at 0, 60, 120, 180 min respectively (reaction volume = 20 μL). Each reaction products were separated on an 8 M urea denaturing 10% PAGE, and imaged with Fx imager after SYBRgreenII staining. M; 10 bp DNA step ladder marker (Promega). Black arrow indicates the mobility of each RNA templates (50 nts). For bands indicated by asterisk and short arrows, see text. Other constant bands were from components of cell-free translation system.

Four kinds of single stranded RNA (ssRNA) of which sequences were different in the 3’-terminal three nucleotides (Figure 2A, underlined) were used as an RNA template. As shown in Figure 2B, when NV3D^pol^ and the RNA template were incubated at 30°C and analyzed on a denaturing PAGE, two reaction products of 50 nts and 90–100 nts were amplified with time. In the case of Temp(GGG-CCC) and Temp(GGG-CCA), RNA templates (50 nts) were amplified as indicated with black arrow in Figure 2B (i, iii) (see Additional file 3: Figure S3A, closed circle and triangle). In the case of Temp(GGG-GGG) and Temp(GGG-UAC), RNA templates were amplified slightly (see Additional file 3: Figure S3A, closed diamond and square). In the case of Temp(GGG-CCC), when the amplified product was treated with ribonuclease S1, it was not digested (shown in Additional file 4: Figure S4). Thus, the amplified products were a double stranded RNA (dsRNA). NV3D^pol^ made a dsRNA from an ssRNA in a primer-independent manner and amplified it. This result is consistent with the report by Rohayem *et al.*[17,18] and Fukushi *et al.*[19]. In addition to the amplification product of 50 nts, amplification products between 90 and 100 nts were observed in the case of Temp(GGG-GGG), Temp(GGG-CCA) and Temp(GGG-UAC) (Figure 2B (ii, iii, iv) indicated by asterisks and arrow heads). We show Additional file 3: Figure S3B as a non-denaturing PAGE version of Figure 2B which was from denaturing PAGE. In Additional file 3: Figure S3B, all the templates ((i) Temp(GGG-CCC), (ii) Temp(GGG-GGG), (iii) Temp(GGG-CCA) and (iv) Temp(GGG-UAC)) gave growing band near 50 bp dsRNA size. For (i), it was confirmed to be actually dsRNA by S1 nuclease treatment (Additional file 4: Figure S4). In Figure 2B, for (i), the growing band was simply 50 nts ssRNA, but for (ii) - (iv), the growing bands were not only 50 nts, but also 90 – 100 nts ssRNA. The possible explanation is as follows: the growing band near 50 bp dsRNA in Additional file 3: Figure S3B (ii) - (iv) corresponds to hairpin RNA (major component) (plus dsRNA (minor component)). These must be self-priming products starting from a short stem-loop at 3’-terminus. In fact in each sequence of (ii) - (iv) we found such a small potential stem-loop at 3’-terminus (shown in Additional file 5: Figure S5), but in the sequence of (i) we did not find any small potential stem-loop at 3’-terminus. This result is consistent with the reports by Belliot *et al.*[20] and Wei *et al.*[22].

**Figure 3:**
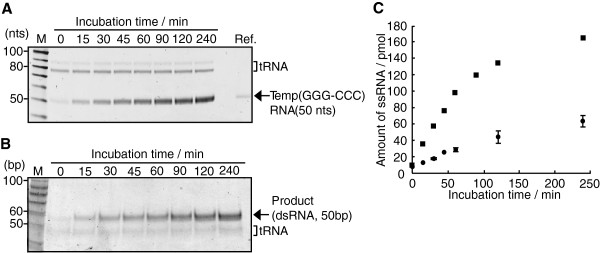
**Isothermal amplification reaction of Temp(GGG-CCC) RNA (initial concentration of 10 or 8 pmol) with NV3D**^**pol **^**(11 or 4 pmol).** Time-course of RNA synthesis reaction with NV3D^pol^. RNA template (10 pmol) was incubated with NV3D^pol^ (11 pmol) at 30°C. The reaction in each aliquot was stopped by adding EDTA, and analyzed on an 8 M urea denaturing 12% PAGE (A) or a non-denaturing 12% PAGE (B). M; 10 bp DNA step ladder (Promega). The RNAs appeared in the 80–90 nts (in a case of **(A)**) or in the 40–50 bp (in a case of **(B)**) mobility were tRNA from the cell-free translation system. **(C)** Closed square: Quantification of each bands in (A) (= the amount of ssRNA). Closed circle: Quantification of template-excess experiments (n = 2) (RNA template 8 pmol and NV3D^pol^ 4 pmol in 32 μL reaction volume). Same lot of the enzyme was used. Quantification of each bands was performed by a calibration curve of Temp(GGG-CCC) RNA (2, 5, 7 and 10 pmol) on the denaturing PAGE.

The time-course of the primer-independent amplification reaction of Temp(GGG-CCC) with NV3D^pol^ was measured and detected on a denaturing PAGE (Figure 3A) or a non-denaturing PAGE (Figure 3B), and quantified (Figure 3C). As shown in Figure 3B, dsRNA of about 50 bp was amplified with time on a non-denaturing PAGE. In the previous studies [17-19], a time-course measurement of amplification reaction was not reported. As shown in Figure 3C closed square, when the amplification reaction was performed with 11 pmol of NV3D^pol^ and 10 pmol of Temp(GGG-CCC) RNA, the RNA template was amplified linearly up to 60 min and reached at plateau near 240 min. On the denaturing PAGE (Figure 3A), about 160 pmol of ssRNA (50 nts) was detected at 240 min, thus NV3D^pol^ amplified 10 pmol of ssRNA to about 80 pmol of dsRNA isothermally in a test tube (16-fold amplification). When 4 pmol of NV3D^pol^ and 8 pmol of Temp(GGG-CCC) RNA were incubated and quantified on the denaturing PAGE, the amplification curve was shown by closed circle in Figure 3C. In the both amplification reaction, the concentration of Temp(GGG-CCC) RNA was constant (0.25 μM). As shown in Figure 3C, the amplification efficiency was depended on the concentration of NV3D^pol^ (closed square versus closed circle). Activity of our enzyme varied between different lots. But within the same lot the activity was fairly reproducible as shown in Figure 3C (closed circle). Thus comparative studies using enzyme from the same lot were meaningful.

### 3’-terminal sequence dependence of initiation of ssRNA replication in a primer-independent manner

Rohayem *et al.*[17] indicated the following two points. First, NV3D^pol^ initiated the replication on an oligo cytidine. Second, NV3D^pol^ added some cytidines at the 3’-terminus of the synthesized strand. From these results, they suggested that NV3D^pol^ prefers a C-stretch for the initiation of replication in primer-independent manner.

**Figure 4:**
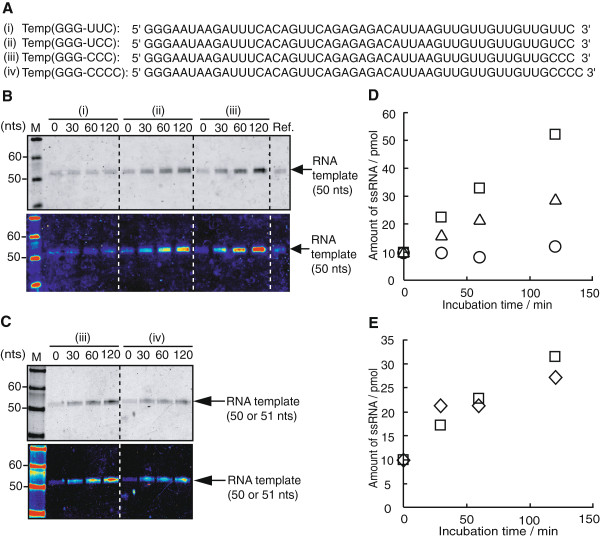
**Effect of the 3’-terminal sequence of template for the initiation efficiency of RNA replication. (A)** The sequence of Temp (GGG-UUC), Temp (GGG-UCC), Temp (GGG-CCC) and Temp (GGG-CCCC). **(B)** and **(C)** NV3D^pol^ (10 pmol) and each RNA templates (10 pmol) were incubated at 30°C and sampled at 0, 30, 60, 120 min (reaction volume = 40 μL). Aliquots were analyzed on an 8 M urea denaturing 10% PAGE and imaged with Fx imager after SYBRgreenII staining. Color-coded images were shown at the bottom. M; 10 bp DNA step ladder marker (Promega). Ref.; Temp (GGG-CCC) RNA. **(D)** and **(E)** Quantification of the amount of RNA strands from the denaturing PAGE (B) and (C), respectively. RNA quantification method was same as in Figure 3. Circle; Temp(GGG-UUC), triangle; Temp (GGG-UCC), square; Temp (GGG-CCC) and diamond; Temp (GGG-CCCC), respectively.

We investigated the number of cytidine for the efficient initiation of replication in primer-independent manner. Temp(GGG-UUC), Temp(GGG-UCC), Temp(GGG-CCC) or Temp(GGG-CCCC) RNA, which have from 1 to 4 cytidine(s) at their 3’-terminus (shown in Figure 4A) and have no small stem-loop at their 3’-terminus, were incubated with NV3D^pol^ at 30°C. The time-course measurements of each case were analyzed on a denaturing PAGE (Figure 4B and C) and quantified (Figure 4D and E). At least two cytidines were necessary for the amplification. And in the case of Temp(GGG-CCC) RNA, the initiation efficiency of replication seemed to be saturated at three cytidines.

**Figure 5:**
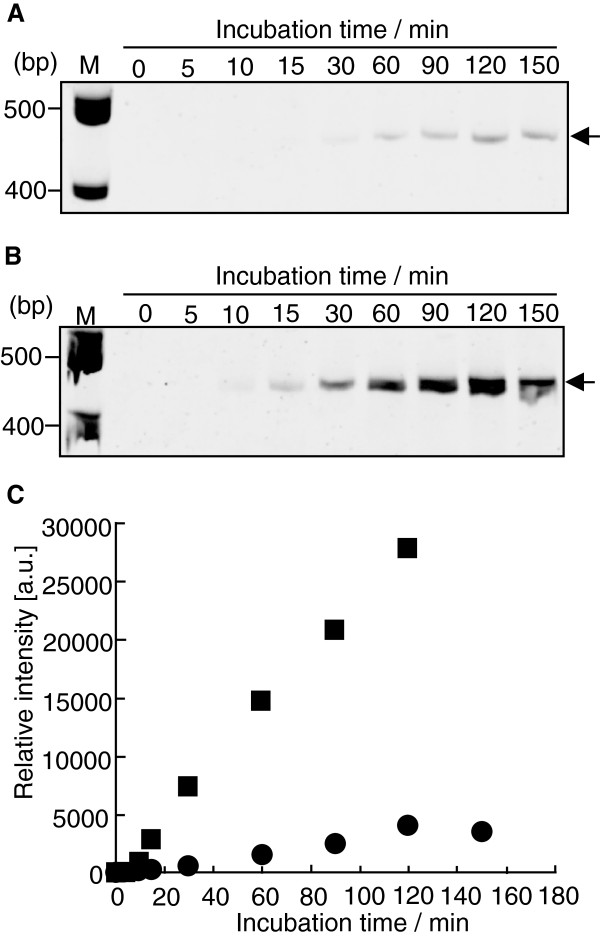
**Effect of the 3’-C-stretch of a long template for the initiation efficiency of RNA replication.** Two kinds of RNA template (TD257-735 **(A)** and TD257-735g734c **(B)** (0.4 pmol) were incubated with NV3D^pol^ (8 pmol), respectively (reaction volume = 20 μL), and analyzed on a non-denaturing 5% PAGE. M; 100 bp DNA ladder marker (Promega). **(C)** The PAGE images were visualized by SYBRgreenII staining and quantified with reference to 0 min as the standard. The band intensities of amplified dsRNA were plotted. Closed circle; TD257-735. Closed square; TD275-735g734c.

We next measured the time-course of amplification reaction using two kinds of 479 nts RNA template, named TD257-735 RNA and TD257-735g734c RNA. These RNA templates (derived from pTD1 expression vector [23]) were not from norovirus genome. Thus the initiation efficiency of these RNA templates is not related to NV3D^pol^ specificity. And the 3’-terminal structure of both templates is predicted to be a free single strand. TD257-735g734c RNA was generated by substituting ---CCGC-3’ at the 3’-terminal region of TD257-735 RNA with ---CCCC-3’. The reaction aliquots were analyzed on a non-denaturing PAGE (Figure 5A and B), and quantified (Figure 5C). By substituting the 3’-terminal sequence to a C-stretch (from ---CCGC-3’ of TD257-735 RNA to ---CCCC-3’ of TD257-735g734c RNA), the amplification efficiency was increased remarkably.

While Rohayem *et al*. [17] showed that NV3D^pol^ does not have an RNA synthesis activity on poly(A)-tail, Fukushi *et al*. [19] indicated that NV3D^pol^ has this activity. In order to re-examine this activity, we synthesized an RNA bearing an A-stretch of 22 nts at the 3’-terminus (named TD257-735-A_22_), and incubated with NV3D^pol^. Our NV3D^pol^ showed a slight activity to poly(A)-tail and the amplified products appeared after 30 min (Additional file 6: Figure S6).

### Terminal nucleotidyl transferase activity and replication of dsRNA

In the reaction using Temp(GGG-CCC), the amplified product, sampled at 240 min of incubation, reaching at plateau, was sequenced. The replicated complementary RNA (RNA(-)) was reverse-transcribed into cDNA using Y-ligation technique shown in Methods. The determined sequences were shown in Table 1. We could observe the addition of 1–3 cytidines at the 3’-terminus of RNA(-). But 50% of the sequenced RNA(-) had no added cytidine at the 3’-terminus. We could also observe the addition of two nucleotides of adenosine or guanosine.

**Table 1:** 3’-terminal sequence of replication products under potential TNT activity

**TNT activity**	**3’-terminal sequence of cRNA**	**Number of clones**
TNT less	3’ CCCUUA--	11
TNT 1 nts (C)	3’ CCCCUUA--	3
TNT 2 nts (CC)	3’ CCCCCUUA--	3
TNT 3nts (CCC)	3’ CCCCCCUUA--	1
TNT 2nts (AA)	3’ AACCCUUA--	1
TNT 2nts (GG)	3’ GGCCCUUA--	1
Deletion, insertion	Others	2

The result of 3’-terminal sequencing of amplified products (n = 22) in the reaction using Temp(GGG-CCC) shown in Figure 3, sampled at 240 min of incubation.

**Figure 6:**
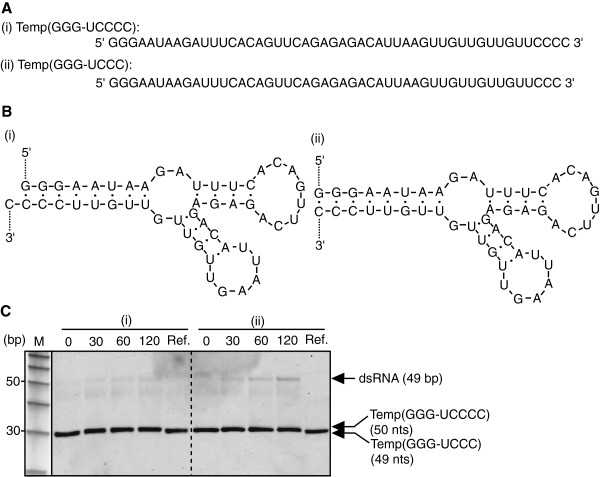
**Effect of the terminal structure of dsRNA for the initiation of RNA replication.** Two kinds of RNA template (10 pmol) (**(i)** Temp(GGG-UCCCC) and **(ii)** Temp(GGG-UCCC) **(A)**), of which secondary structures were predicted by Mfold **(B)**, were incubated with NV3D^pol^ (4 pmol), respectively (reaction volume = 40 μL), and analyzed on a non-denaturing 10% PAGE. The PAGE image was visualized by SYBRgreenII staining **(C)**. M; 10 bp DNA step ladder (Promega). Ref; both RNA templates were electrophoresed as references. Mfold predicted many other structures, but the stem-end (3’end and 5’end hybrid) structures were almost always conserved, namely, the probability of other 3’ structures (for (i), first appearance as #7 stable structure (ΔG of #1, #2,---,and -#7 structures were -12.83, -11.08, -10.49, -10.10, -9.39, -8.52, and -7.99 kcal/mol, respectively) was less than 0.03% .

It was reported that the sticky end formed by this TNT activity is favourable for the next round replication of the dsRNA [17]. We re-examine this point. Two kinds of RNA template (shown in Figure 6A and B) were incubated with NV3D^pol^ at 30°C and analyzed on non-denaturing PAGE. The secondary structures of these two RNA templates at 30°C predicted by Mfold [24] were shown in Figure 6B. Mfold predicted many other structures, but the stem-end (3’end and 5’end hybrid) structures were almost always conserved, namely, the probability of other 3’ structures was less than 0.03%. Thus these two RNAs are able to be models of dsRNAs made in the first cycle replication of an ssRNA template (e.g. Temp(GGG-CCC) with or without TNT activity. As shown in Figure 6C, the band at about 50 bp was amplified in the case of Temp(GGG-UCCC), but it was not in the case of Temp(GGG-UCCCC). It was suggested that NV3D^pol^ replicates a blunt end dsRNA preferably rather than a sticky end dsRNA.

### Isothermal amplification of a long RNA

**Figure 7:**
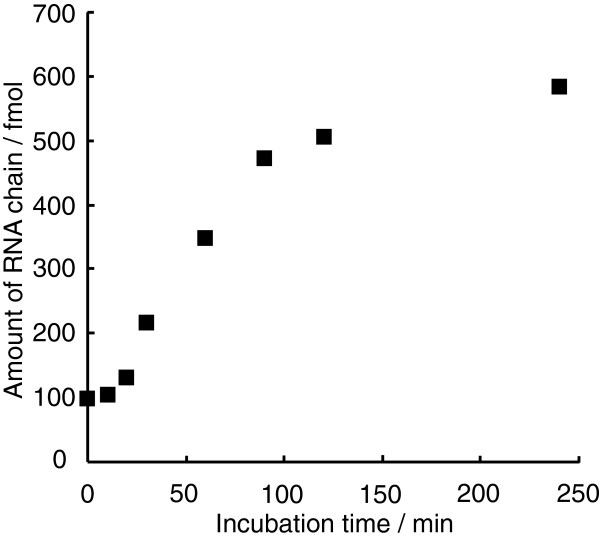
**A time-course of primer-independent amplification reaction of RNA (479 nts) with NV3D**^**pol**^**.** In the reaction mixture (reaction volume = 40 μL), the amount of NV3D^pol^ (2 pmol) was twenty-fold of RNA template (TD257-735g734c, 0.1 pmol). Each aliquot was added with EDTA to stop the reaction, analyzed on a non-denaturing 5% PAGE, visualized by SYBRgreenII staining, and quantified. The vertical axis represents the amount of total RNA strands (ssRNA + dsRNA × 2).

To observe the exponential phase in the isothermal amplification of comparatively long RNA with NV3D^pol^, TD257-735g734c RNA and NV3D^pol^ were incubated at the ratio of 1 : 20 in a reaction tube. Each aliquot was analyzed on a non-denaturing PAGE followed by the staining with SYBRgreenII and quantified (Figure 7). The initial RNA template (ssRNA) of 100 fmol was amplified to 250 fmol of dsRNA plus 100 fmol ssRNA in 240 min (6-fold amplification). The initial apparently exponential phase was observed reproducibly.

### Primer extension replication

**Figure 8:**
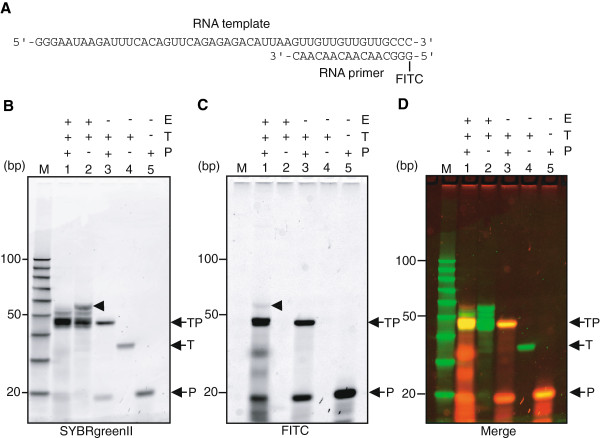
**Primer extension replication with NV3D**^**pol**^**. (A)** Schematic illustration of the template-primer hybridization. NV3D^pol^ (the amount was not detected) was incubated with template-primer hybrid (initial concentration of 5 pmol and 10 pmol, respectively) (lane1) or RNA template (initial concentration of 5 pmol) (lane2) (reaction volume = 20 μL), analyzed on a non-denaturing 10% PAGE, visualized by SYBRgreenII staining **(B)** or FITC fluorescence **(C)**. Both images were merged **(D)**. E, T, P and TP indicate NV3D^pol^, RNA template, RNA primer and template-primer hybrid, respectively. +/- corresponds to the presence or absence of substrates. M; 10 bp DNA step ladder (Promega).

Rohayem *et al.*[17] showed the primer extension replication with NV3D^pol^, but Fukushi *et al.*[19] suggested the enzyme does not have such an activity. We examined the activity as follows. A RNA template, Temp(GGG-CCC), hybridized with a RNA primer (16 nts) at the 3’-terminal region was incubated with NV3D^pol^ and NTPs. Here, the 5’-terminus of RNA primer was modified with FITC to trace whether NV3D^pol^ elongates the RNA primer. That is, 5 pmol RNA template and 10 pmol RNA primer were hybridized, and then mixed with 4 pmol of NV3D^pol^ and incubated at 30°C. As a control, primer-independent RNA synthesis reaction of Temp(GGG-CCC) RNA with NV3D^pol^ was performed at the same time. The reaction products were analyzed on a non-denaturing PAGE or an 8M urea denaturing PAGE, and imaged (shown in Figure 8 and Additional file 7: Figure S7). The dsRNA generated with NV3D^pol^ was indicated by arrowhead in Figure 8B, lane2. In Figure 8C, lane1, the band indicated with arrowhead, which was detected with FITC fluorescence was about same molecular weight with dsRNA generated in a primer-independent manner (detected in lane 2 in Figure 8B, and not detected in lane2 in Figure 8C). Therefore, this product was a dsRNA generated in the primer-dependent manner with NV3D^pol^.

## Discussion

### Initiation reaction

When a small stem-loop structure can be formed at 3’-terminus, the self-priming is more favorable initiation reaction for an ssRNA template than the primer-independent manner as seen in Figure 2B and Additional file 3: Figure S3B. For (iii), 3’-terminal -CA forms a hairpin stem with -UG- at upstream. For (iv), 3’-terminal -AC forms a hairpin stem with -GU- at upstream. As the template contains -GUU- repeat at 3’-terminal region, several hybridization sites can be expected. The fact that there was only one band of double length shows NV3D^pol^ recognizes small loop size (3 for (iii), 4 for (iv)).

In this context we comment the self-priming case by Belliot *et al.*[20], and Wei *et al.*[22]. They used an RNA template of which 3’-terminal sequence is ---GAUCCAAGCUUACGU*ACGCG*-3’ (the sequence showed in italics is a part of cutting site of *Mlu*I restriction enzyme) and got a double length replication product. They explained it is the result of the back-priming at *Mlu*I palindrome. This explanation is not acceptable based on thermodynamic considerations. We propose another explanation that it was the result of self-priming starting from a small stem-loop (loop size = 4 nts, stem size = 2 bp) made of ---CGUACGCG-3’.

Fullerton *et al.*[25] investigated the initiation reaction of RNA replication with Sapovirus (family with norovirus) replicase using 2259 nts template of which 3’-terminal sequence is ---UUGGAGCCAUUGCCCUCCAU-3’. They did not get a double length product and concluded the initiation by the primer-independent manner. In fact a potential 3’-terminal stem-loop has loop size eight (Additional file 5: Figure S5B (b)). This is not a small size and self-priming can hardly proceed. This results support our conclusion that when there is a small stem-loop (loop size 3–4, stem size 2) structure exists at 3’-terminus, the self-priming is the most preferable initiation reaction for an ssRNA template.

There were at least two amplification reaction products of the template Temp(GGG-GGG) (Figure 2 (ii); indicated with asterisk). Length of them was between 92 and 100 nts. If we consider a small stem-loop made with GG:UU stem, the length of self-priming product would be 91, 88, or 85 nts according to the loop size 5, 8, or 11 nts, respectively (Additional file 5: Figure S5A (ii)). Here we neglect loop size 2 nts, because it is very unstable because of the steric hindrance and the hairpin thermodynamics. They are probably originated from C and CCC addition to 3’-terminus with TNT activity of the enzyme. The products can make small stem-loops of GUUGGGGC and GGGGCCC which can make 94 nts and 99 nts product, respectively (Additional file 5: Figure S5A (ii)).

When there exists no small stem-loop structure at 3’-terminus and 3’-terminal sequence has C-stretch, the primer-independent initiation proceeds as shown in Figure 2B (i) and in the above mentioned case by Fullerton *et al.*[25]. Rohayem *et al.*[17] also pointed out that NV3D^pol^ prefers C-stretch at 3’-terminus for the replication initiation of the primer-independent manner. Importance of the C-stretch was confirmed also the experiment using a long template of which sequence had no relation to norovirus genome. TD257-735g734c template showed six times larger amplification than TD257-735 template. The former is a point mutant of the latter and the 3’-terminal sequence is ---UCCCC and ---UCCGC, respectively. They have no stable small stem loop at 3-terminus.

Although Rohayem *et al*. [17] indicated that NV3D^pol^ did not have an RNA synthesis activity on poly(A)-tail, we showed that NV3D^pol^ initiated replication reaction on A-stretch at the 3’-terminus of TD257-735-A_22_ template (Additional file 6: Figure S6). The RNA synthesis activity on A-stretch, however, was considerably lower than the case of TD257-735g734c template. Therefore, the initiation of primer-independent RNA replication reaction by NV3D^pol^ prefers C-stretch at the 3’-terminus rather than A-stretch or poly(A)-tail. The differences between Rohayem’s system and ours are the amino acids sequence of NV3D^pol^, the length of poly(A)-tail, and enzyme concentration used. On the other hand, Fukushi *et al*. [19] already reported that NV3D^pol^ has the initiation activity of primer-independent RNA replication on poly(A)-tail. We supposed that the actual 3’-terminal sequence of RNA template used in Fukushi’s work was ---(A_30_)UGCGC.

It is reported that Qβ replicase also favours C-stretch. The initiation efficiency is high when 3’-terminal sequence is ---CCC-3’ or ---CCA-3’. The efficient parasite RQ RNA has also the sequence ---CCC-3’. By the way, we have not yet observed any parasite sequence in amplification with NV3D^pol^.

In the case of a blunt-end double-stranded RNA, the breathing of terminal double helical region makes the enzyme access to CC-stretch at 3’-terminus and start to replication in the primer-independent manner. To investigate whether NV3D^pol^ initiates the replication to dsRNA which has a sticky-end preferably rather than the blunt-end [17], we prepared two kinds of RNA template, Temp(GGG-UCCCC) and Temp(GGG-UCCC) shown in Figure 6A. The secondary structure predicted by Mfold (Figure 6B) shows that the former mimics the terminal structure of dsRNA added a cytidine by TNT activity and the latter mimics the terminal structure of a blunt-end. As shown in Figure 6C, the band at about 50 bp were amplified in the case of Temp(GGG-UCCC), but not in the case of Temp(GGG-UCCCC). This indicates that NV3D^pol^ might initiate the replication from the blunt-end cytidines at the 3’-terminus preferably rather than from sticky-end cytidines. We investigated only about the addition of one cytidine. Although the addition of three cytidines was rare (Table 1), if dsRNA which has three cytidines overhung end were used, the initiation efficiency of replication might alter.

The initiation of the extension reaction of a long primer is not favourable for the enzyme as shown in Figure 8. Rohayem *et al.*[17] reported primer-dependent replication, but Fukushi *et al.*[19] reported their NV3D^pol^ had no activity of primer extension. Our gene was a gift of Fukushi’s group. Thus they might miss the faint band.

### Elongation reaction

The self-priming reaction at a small stem-loop at 3’-terminus is very active, but the extension activity of a long primer is very poor. Thus once the enzyme dissociates from the template-polymerizing chain hybrid, it is not easy for the enzyme to bind again. On the other hand as shown in Figure 5, the native state PAGE of the amplified product (479 bp) shows a single sharp band without smear. Thus we can draw the conclusion that the processivity of NV3D^pol^ is very high.

NV3D^pol^ can perform also the chain dissociation replication smoothly as shown in Figure 5, even in the case of a template having GC-rich regions (Figure 7 and Additional file 8: Table S1(c)). The amplification products must be double stranded and amplification proceeded several times. The dissociated single stranded chain was converted to double strand in a primer-independent manner not so rapidly, because the band of ssRNA was visible on the non-denaturing PAGE (Additional file 3: Figure S3B).

### Termination reaction (TNT activity)

Fukushi *et al.*[19] reported that their NV3D^pol^ had no TNT activity. Gene of NV3D^pol^ of our study is a gift from the laboratory in which their study was performed. Our results rather accords qualitatively with the report by Rohayem *et al.*[17,18]. They reported TNT activity of NV3D^pol^ added at least four cytidines, but we observed -C, -CC and -CCC addition. We also observed about half of products had no addition of C. The C-stretch (corresponding to G stretch on the template) at 3’-terminus in our replicate chain may affect the reduced number of addition. We also observe addition of A and G, which accords with the reports by Rohayem *et al.*[18] and Fullerton *et al.* (Sapovirus 3D^pol^) [25].

It was also reported that Moloney murine leukemia virus (MMLV) adds some deoxycytidines at the 3’-terminus of reverse transcribed DNA, and switches the template utilizing this overhung dC-stretch [26]. If NV3D^pol^ has the same template-switching activity as MMLV, the band corresponding to about 100 nts, observed in the replication reaction of Temp(GGG-GGG) RNA (Figure 2 (ii)), might be a denatured form of a tandem dimer dsRNA made through this activity rather than a double length dsRNA which was the product of replication from a self-priming product. Based on the fact that initiation efficiency of primer-independent manner at 3’-terminus GGG is significantly lower than self-priming with a small stem-loop, this explanation for 100 nts band is not acceptable.

### Autocatalytic replication

In the non-self-priming and primer-independent replication shown in Figure 3B and Figure 7, the initial template ssRNA was amplified by 16-fold and 6-fold by 240 min with NV3D^pol^, respectively. In the former, the shape of amplification curve showed two phases; linear amplification phase up to 90 min and plateau phase near 240 min, because the enzymatic activity of NV3D^pol^ is limited by about 120 min, as indicated in previous reports [18]. The autocatalytic replication should be realized in this case, but the exponential growth phase was not observed because the enzyme was not excess over the initial template and the linear phase can be explained by the full turnover of enzyme action. On the other hand, in the case of Figure 7, the enzyme was excess over the initial template. And the growth curve of RNA shows three phases; the initial nonlinear phase, the apparent linear phase and the plateau phase. The final phase was realized by the same reason as in the case of Figure 3B. The apparent linear phase was not originated from the full turnover of enzyme action because the enzyme was vastly excess over the existing RNA template. This apparent linear phase may be explained by attenuated exponential growth caused by deactivating replicase. The initial nonlinear phase may be explained two fold; the initial part of the exponential phase or the reaction curve of the two- (or multi-) step reaction. The isothermal amplification of RNA with Qβ replicase also showed exponential growth of RNA under the similar condition [16].

### A possible model of molecular mechanism of the isothermal amplification of RNA with NV3D^pol^

**Figure 9:**
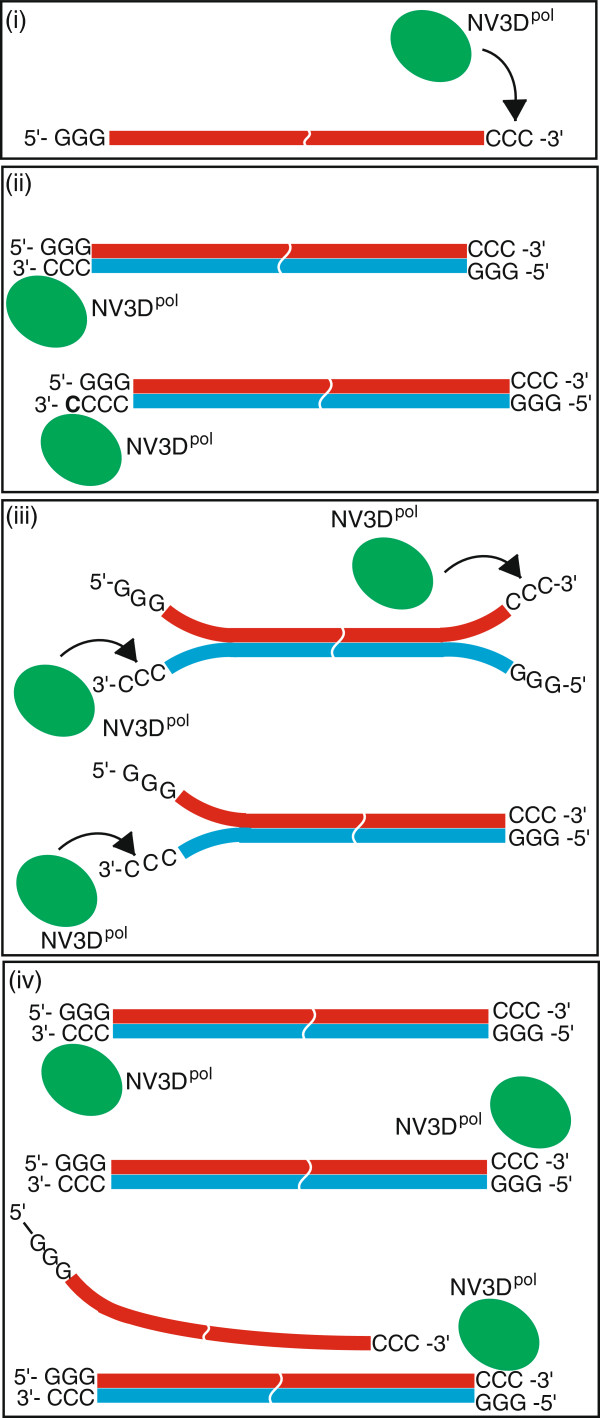
**A model scheme of isothermal RNA amplification with NV3D**^**pol**^**. (i)** NV3D^pol^ interacts to the CCC sequence at the 3’-terminus of RNA template (RNA(+)), and **(ii)** made dsRNA (RNA(+) - RNA(-)). Sometimes the enzyme adds cytidine(s) at the 3’-terminus of RNA(-) by TNT activity. **(iii)** NV3D^pol^ interacts to the breathing CCC sequence. **(iv)** NV3D^pol^ replicates the dsRNA with displacing the RNA strand. The displaced RNA strand is recycled as a RNA template.

Rohayem *et al.*[17] showed a protein-primer model of norovirus genomic RNA replication in an infected host cell. Uridylated VPg protein binds to poly(A)-tail and begins replication starting from the U. Our interest is not the viral infection process, but application to *in vitro* RNA amplification for an *in vitro* evolution. Based on the results of this study together with preceding reports, we propose the following model for the isothermal RNA amplification with NV3D^pol^*in vitro* (Figure 9) for templates having limited terminal sequences. NV3D^pol^ recognizes 3’-terminus -CCC of the template ssRNA and begins the synthesis of the complementary strand (without primer). Thus, in order to sustainable replication, 5’-terminus of the template must be –GGG. NV3D^pol^ prefers, however, a small stem-loop structure at the 3’-terminus and begins RNA synthesis in the self-priming manner. Thus, both terminal sequences should not make such a small stem-loop. At the end of template synthesis, NV3D^pol^ sometimes adds several C using its TNT activity. Thus, 3’-terminus of the replicated strand (RNA(-)) has a C-stretch just as the template (RNA(+)) or sometimes a slightly longer C-stretch, because NV3D^pol^ recognizes the C-stretch at 3’-terminus of dsRNA, often accessible when it is breathing, and begins synthesis of the next generation strand using the chain dissociation replication activity.

## Conclusions

In this study, it was confirmed that NV3D^pol^, prepared with cell-free protein synthesis system, was able to perform the isothermal amplification of RNA (even dsRNA) *in vitro*. But the initiation efficiency of replication is dependent on the 3’-terminal sequence. And we have to use the 3’-terminal sequence which does not make a small stem-loop, in order to avoid a parasitic self-priming product. Thus, NV3D^pol^ can be used as an RNA replicase in *in vitro* RNA evolution with the RNA of special terminal sequences. NV3D^pol^, which is a single chain protein, is a candidate of a model protein in *in vitro* autonomous protein evolution in the form of an *in vitro* virus [27].

## Methods

### Preparation of NV replicase (NV3D^pol^)

Plasmid pVL3Dwt (GenBank: AB039782 [19]) harbouring NV3D^pol^ gene was kindly provided from BML Inc. PCR was performed with KOD-plus- DNA polymerase (TOYOBO) and the PCR products were purified with QIAquick PCR Purification Kit (QIAGEN). NV3D^pol^ gene was amplified from pVL3Dwt by PCR using prNV3Dstart(+) 5′-ATGGGAGGTGACGACAAGGGC-3′ and prNV3Dstop(-) 5′-TTATTCGACGCCATCTTCATTCACA-3′. NV3D^pol^ gene was modified with the coding region for Strep-tag II sequence (WSHPQFEK) [21] using prNV3Dstart(+) and prNV3D-strep(-) 5′-GCATCGACTCC*TTACTTTTCAAACTGCGGATGGCTCCA*TTCGACGCCATCTTCATTC-3′ (the sequence showed in italics indicates stop codon and Strep-tag II coding sequence) by PCR. At the downstream of the stop-codon, *KpnI* restriction site was added using prNV3Dstart(+) and prStrep-kpn1(-) 5′-GG*GGTACCTTA*CTTTTCAAACTGCGGATGGCTCC-3′ (the sequence showed in italics indicates *KpnI* cutting site) by PCR. Then the PCR product was digested with *KpnI* (TaKaRa) and integrated into the pTD1 expression vector (SHIMADZU Biotech) [23] according to the manufacture’s instruction. The recombinant plasmid was named pTD-NV3D^pol^-strep. NV3D^pol^-strep expression construct DNA was amplified using prTD161-179 (5′-GCAGATTGTACTGAGAGTG-3′) and prTD845-827 (5′-GGAAACAGCTATGACCATG-3′), and transcribed *in vitro* with RiboMAX^TM^ Large Scale RNA production system-T7 (Promega). The transcript, named NV3D^pol^-strep mRNA was purified with NICK column (GE Healthcare). NV3D^pol^-strep mRNA was translated with Transdirect *insect cell* cell-free protein synthesis kit (SHIMADZU Biotech) [28] according to the manufacture’s instruction.

The translated product was purified with Strep-tactin Superflow plus (QIAGEN). The strep-tag II modified NV3D^pol^ was bound onto Strep-tactin Superflow plus resin, pre-equilibrated with binding buffer (50 mM Tris–HCl [pH 8.0], 300 mM NaCl). The bound protein was washed with the binding buffer and eluted with elution buffer (50 mM Tris–HCl [pH 8.0], 300 mM NaCl, and 2.5 mM desthiobiotin (Sigma)). The eluted protein was then enriched and buffer-exchanged with buffer A (25 mM Tris–HCl [pH 8.0], 100 mM NaCl, 5 mM MgCl_2_, and 1 mM β-mercaptoethanol) with Microcon YM-50 column (Millipore), and stored at -80°C. In the enrichment step using Microcon YM-50 column, bovine serum albumin (BSA) (TaKaRa) was used as a carrier protein to avoid the non-specific adsorption of NV3D^pol^ to the column. The amount of NV3D^pol^-strep was quantified on sodium dodecyl sulfate poly-acrylamide gel electrophoresis (SDS-PAGE) referring a calibration curve with BSA.

### Preparation of RNA templates

Sequences of RNA template used in this study were summarized in Additional file 9: Table S1 (a). Temp(GGG-CCC) DNA was generated by PCR using Temp(GGG-CCC) (5′-GCCAGTCGCCTGCAG*TAATACGACTCACTATA*GGGAATAAGATTTCACAGTTCAGAGAGACATTAAGTTGTTGTTGTTGCCC-3′) (underline indicates T7 Φ6.5 promoter sequence), prT7tempGGG + (5′-GCCAGTCGCCTGCAGTAATACGACTCACTA-3′) and prTempCCC- (5′-GGGCAACAACAACAACTTAATGTC-3′). Other DNA templates, Temp(GGG-GGG), Temp(GGG-CCA) Temp(GGG-UAC), Temp(GGG-UUC), Temp(GGG-UCC), Temp(GGG-GCCCC), Temp(GGG-UCCCC) and Temp(GGG-UCCC) DNA, were generated as same as Temp(GGG-CCC) DNA with the primer listed in Additional file 9: Table S1 (b). All PCR amplified DNAs were purified with QIAquick PCR Purification kit and *in vitro* transcribed with RiboMAX™ Large Scale RNA production system-T7. TD257-735 RNA were also *in vitro* transcribed from the PCR amplified DNA (the sequence was shown in Additional file 9: Table S1 (c)) using pTD1 [23], prTD161-179 and prTD735-714 (5′-GCGGATAATATTTTGAACGACG-3′). TD257-735g734c RNA was made from TD257-735 by replacing 734^th^ guanosine to cytidine using prTD735-714g734c (5′-GGGGATAATATTTTGAACGACG-3′) in PCR. TD257-735-A_22_ was made from TD257-735 by adding 22 nts of adenine at the 3’-terminus using prTD757-733 (5′-TTTTTTTTTTTTTTTTTTTTTTGCG-3′) in PCR. All RNA templates were purified on denaturing PAGE and quantified by measuring absorbance at 260 nm.

### RNA primer

RNA primer (5′-GGGCAACAACAACAAC-3′) modified with FITC at the 5’-terminus was purchased from Japan Bio Service Inc.

### *In vitro* RNA replication or amplification with NV3D^pol^-strep

RNA replication or amplification was performed with purified NV3D^pol^-strep and *in vitro*-transcribed RNA template in a reaction buffer (50 mM Hepes-KOH [pH 7.0], 3 mM MnCl_2_, 4 mM DTT, 0.4 mM rNTPs, and 40 U/μL of RNasin ribonuclease inhibitor plus (Promega)) at 30°C. Reaction products were subjected to denaturing or non-denaturing PAGE followed by SYBRgreenII (Lonza) staining and visualized on Pharos Fx imager (Bio-Rad). Here, the non-denaturing PAGE was performed with 5 or 10% acrylamide, 0.625% bisacrylamide and TBE buffer at 20°C. The denaturing PAGE was performed with 8 M urea plus the same constituent of non-denaturing PAGE at 65°C.

### Sequencing of 3’-terminus of amplified RNA

To detect the addition of nucleotides at the 3’-terminus of replicated RNA (RNA(-)) by TNT activity of NV3D^pol^, we could not use a normal sequencing method using a primer which hybridizes 3’-terminal region of the sample RNA. Thus we sequenced the full length of RNA(-) applying the Y-ligation method [29], as follows (shown in Additional file 9: Figure S8). After the incubation of 240 min in the RNA amplification with NV3D^pol^-strep, the reaction solution was desalted with Micro bio-spin column 30 (Bio-Rad), and ligated with 5’-phosphorylated Y-adapter (5′-CAAAGGGAATAAGATTTCACAGTTCAGAGCTTAGATAATACGACTCACTATAGGGTTAAC-3′) by Y-ligation method. The ligated product was hybridized with prT7g10SD-NV3D(-) (5′-CCTTGTCGTCACCTCCCATGGATATATCTCCTTCTTAAAGTTAACCCTATAGTGAGTCGTATTA-3′) and reverse transcribed with Avian myelobastosis virus (AMV) reverse transcriptase (Promega). The reverse transcribed product was purified on denaturing PAGE, and amplified by PCR using prTempCCC- and prSeq (5′-CCTTGTCGTCACCTCCCA-3′) as primers. TA cloning was performed with pGEM-T easy vector system (Promega). Sequencing was performed by Operon Inc. (Tokyo).

## Competing interests

The authors declare that they have no competing interests.

## Authors’ contributions

HA and YH designed the study, analyzed the data and wrote the paper. HA performed the experiments. NN, MS and KN analyzed the data. All authors contributed to the manuscript and have read and approved the final manuscript.

## Supplementary Material

Additional file 1: Figure S1 Amino acid sequence comparison of NV3D^pol^ proteins used in Rohayem’s report (A), in Fukushi’s report (B) and in this report (C). The Amino acid sequence homologies between (A) and (B), (B) and (C), (A) and (C) were 90.4, 99.2, 89.6%, respectively. Amino acid substitutions were indicated in bold letters. Click here for file

Additional file 2: Figure S2 Primer-independent RNA amplification reaction of Temp(GGG-CCC) RNA with NV3D^pol^ under the excess BSA condition (lane 3 and 4). Temp(GGG-CCC) RNA (5 pmol) was incubated with NV3D^pol^ (the amount was not detected) and sampled at 0 and 120 min. In the case of excess BSA +, 500 ng of BSA was added to the standard reaction mixture (BSA -). M1; 10 bp DNA step ladder (Promega). M2; DynaMarker dsRNA ladder (BioDynamics Laboratory Inc.). Ref.; Temp(GGG-CCC) RNA. Click here for file

Additional file 3: Figure S3 Supplements to Figure 2. (A) Quantification of amplification of 50 nts ssRNA in Figure 2B (8M urea denaturing 10% PAGE). Closed circle; (i) Temp (GGG-CCC), closed diamond; (ii) Temp (GGG-GGG), closed triangle; (iii) Temp (GGG-CCA), closed square; (iv) Temp (GGG-UAC). (B) Non-denaturing PAGE. NV3D^pol^ (5 pmol) was incubated with (i) Temp (GGG-CCC), (ii) Temp (GGG-GGG), (iii) Temp (GGG-CCA) or (iv) Temp (GGG-UAC) (5 pmol each), and sampled at 0, 60, 120, 180 min respectively (reaction volume = 20 μL), which is the same reactions shown in Figure 2, Each reaction aliquots were analysed on a non-denaturing 10% PAGE, and imaged with Fx imager after SYBRgreenII staining. M; 10 bp DNA step ladder marker (Promega). Lot of the enzyme was different from the experiment of Figure 2. Click here for file

Additional file 4: Figure S4 S1 nuclease treatment of the amplification product of Temp(GGG-CCC) . Temp(GGG-CCC) RNA (5 pmol) and NV3D^pol^ (the amount was not detected) were incubated at 30°C for 120 min. The reaction was stopped with adding EDTA, followed by PCI (phenol/chloroform/isoamylalchol = 25 : 24 : 1) and CIA (chloroform/isoamylalchol = 24 : 1) extraction, and ethanol precipitation. Then the pellet was resuspended with 1 × S1 nuclease buffer (TaKaRa) and incubated with 13.5 U/μL of S1 nuclease (TaKaRa) at 37°C for 15 min. Each aliquot was added with EDTA to stop the reaction, and analyzed on an 8M urea denaturing 10% PAGE (A) or a non-denaturing 10% PAGE (B), visualized by SYBRgreenII staining. M1; 10 bp step DNA ladder (Promega). M2; DynaMarker dsRNA ladder (BioDynamics Laboratory Inc.). White headarrows indicated in lane 2 were the nucleic acids from cell-free protein synthesis system (e.g. tRNAs). Click here for file

Additional file 5: Figure S5 Potential small stem-loop structures of 3’-terminus of RNA templates. (A) 3’-terminal sequence and potential small stem-loop structure of Temp(GGG-GGG) (ii), Temp(GGG-CCA) (iii) and Temp(GGG-UAC) (iv). Asterisks indicate the hybridization points between 3’-terminus. In (ii), the potential small stem-loop in the case of addition of 1 - 3 cytidine(s) on 3’-terminus were shown. (B) Potential small stem-loop structures of 3’-terminal sequence used in the previous reports ((a); [20,22], (b); [25]). Click here for file

Additional file 6: Figure S6 Effect of poly(A)-tail for the initiation efficiency of RNA replication. TD257-735-A_22_ RNA (0.4 pmol) were incubated with NV3D^pol^ (4 pmol) (reaction volume = 20 μL) and analyzed on a non-denaturing 5% PAGE. M; 100 bp DNA ladder marker (Promega). In our PAGE condition, dsDNA 400 bp marker (Promega) corresponds to dsRNA 430bp marker (BioDynamics Laboratory Inc.). Click here for file

Additional file 7: Figure S7 Supplement to Figure 8. Denaturing PAGE analysis. Experimental conditions are the same as in Figure 8, except the final analysis step, namely, analyzed on an 8M urea denaturing 10% PAGE, visualized by SYBRgreenII staining (A) or FITC fluorescence (B). Both images were merged (C). Arrowheads indicate the elongate primer (lane 1) and the primer-independent RNA synthesis product (lane 2), respectively. E, T, P and TP indicate NV3D^pol^, RNA template, RNA primer and template-primer hybrid, respectively. +/- corresponds to the presence or absence of substrates. M; 10 bp DNA step ladder (Promega). Click here for file

Additional file 8: Table S1 Sequences of RNA templates **(a)**, DNA oligomers **(b)** used in this work. DNA sequence of 161-757 region in pTD1 **(c)**. Underline indicates T7 ϕ 6.5 promoter sequence. Click here for file

Additional file 9: Figure S8 Schematic illustration of sample preparation for the 3’-terminus sequencing of RNA. As described in Materials and Methods, the reaction procedure is as follows; (1) the replicated RNA (RNA (-), shown in red) was ligated with an adapter DNA (shown in blue) hybridized at the 3’-terminal region of RNA(-) by the Y-ligation method. (2) The ligation product was reverse-transcribed with RT primer (green arrow) to cDNA, (3) and the cDNA was amplified by PCR, cloned and sequenced. Click here for file

## References

[B1] Haruna I Spiegelman S Autocatalytic synthesis of viral RNA in vitro Science 1965 150 884 886 10.1126/science.150.3698.884 5835788

[B2] Biebricher CK Eigen M Luce R Product analysis of RNA generated de novo by Qβ replicase J Mol Biol 1981 148 369 390 10.1016/0022-2836(81)90182-0 7310872

[B3] Biebricher CK Eigen M Luce R Kinetic analysis of template-instructed and *de novo* RNA synthesis by Qβ replicase J Mol Biol 1981 148 391 410 10.1016/0022-2836(81)90183-2 6273581

[B4] Guatelli JC Whitfield KM Kwoh DY Barringer KJ Richman DD Gingeras TR Isothermal, in vitro amplification of nucleic acids by a multienzyme reaction modelled after retroviral replication Proc Natl Acad Sci USA 1990 87 1874 1878 10.1073/pnas.87.5.1874 2308948 PMC53586

[B5] Compton J Nucleic acid sequence-based amplification Nature 1991 350 91 92 10.1038/350091a0 1706072

[B6] Breaker RR Joyce GF Emergence of a replicating species from an *in vitro* RNA evolution reaction Proc Natl Acad Sci USA 1994 91 6093 6097 10.1073/pnas.91.13.6093 7517040 PMC44144

[B7] Mills DR Peterson RI Spiegelman S An extracellular darwinian experiment with a self-duplicating nucleic acid molecule Proc Natl Acad Sci USA 1967 58 217 224 10.1073/pnas.58.1.217 5231602 PMC335620

[B8] Ekland EH Bartel DP RNA-catalysed RNA polymerization using nucleoside triphosphates Nature 1996 382 373 376 10.1038/382373a0 8684470

[B9] Johnston WK Unrau PJ Lawrence MS Glasner ME Bartel DP RNA-catalyzed RNA polymerization: accurate and general RNA-templated primer extension Science 2001 292 1319 1325 10.1126/science.1060786 11358999

[B10] Nemoto N Miyamoto-Sato E Husimi Y Yanagawa H In vitro virus: Bonding of mRNA bearing puromycin at the 3’-terminal end to the C-terminal end of its encoded protein on the ribosome in vitro FEBS Lett 1997 414 405 408 10.1016/S0014-5793(97)01026-0 9315729

[B11] Roberts RW Szostak JW RNA-peptide fusions for the *in vitro* selection of peptides and proteins Proc Natl Acad Sci USA 1997 94 12297 12302 10.1073/pnas.94.23.12297 9356443 PMC24913

[B12] Fodor SP Read JL Pirrung MC Stryer L Lu AT Solas D Light-directed, spatially addressable parallel chemical synthesis Science 1991 251 767 773 10.1126/science.1990438 1990438

[B13] Blumenthal T Carmichael GG RNA replication: function and structure of Qβ-replicase Annu Rev Biochem 1979 48 525 548 10.1146/annurev.bi.48.070179.002521 382992

[B14] Barrera I Schuppli D Sogo JM Weber H Different mechanisms of recognition of bacteriophage Qβ plus and minus strand RNAs by Qβ replicase J Mol Biol 1993 232 512 521 10.1006/jmbi.1993.1407 8345521

[B15] Munishkin AV Voronin LA Ugarov VI Bondareva LA Chetverina HV Chetverin AB Efficient templates for Qβ replicase are formed by recombination from heterologous sequences J Mol Biol 1991 221 463 472 10.1016/0022-2836(91)80067-5 1717699

[B16] Urabe H Ichihashi N Matsuura T Hosoda K Kazuta Y Kita H Yomo T Compartmentalization in a water-in-oil emulsion repressed the spontaneous amplification of RNA by Qβ replicase Biochemistry 2010 49 1809 1813 10.1021/bi901805u 20108973

[B17] Rohayem J Robel I Jäger K Scheffler U Rudolph W Protein-primed and de novo initiation of RNA synthesis by norovirus 3D^pol^ J Virol 2006 80 7060 7069 10.1128/JVI.02195-05 16809311 PMC1489054

[B18] Rohayem J Jäger K Robel I Scheffler U Temme A Rudolph W Characterization of norovirus 3D^pol^ RNA-dependent RNA polymerase activity and initiation of RNA synthesis J Gen Virol 2006 87 2621 2630 10.1099/vir.0.81802-0 16894201

[B19] Fukushi S Kojima S Takai R Hishino BF Oka T Takeda N Katayama K Kageyama T Poly(A)- and primer-independent RNA polymerase of *norovirus* J Virol 2004 78 3889 3896 10.1128/JVI.78.8.3889-3896.2004 15047805 PMC374273

[B20] Belliot G Sosnovtsev SV Chang K Babu V Uche U Arnold JJ Cameron CE Green KY Norovirus proteinase-polymerase and polymerase are both active forms of RNA-dependent RNA polymerase J Virol 2005 79 2393 240 10.1128/JVI.79.4.2393-2403.2005 15681440 PMC546540

[B21] Skerra A Schmidt TGM Use of the *Strep-* tag and streptavidin for detection and purification of recombinant proteins Methods Enzymol 2000 326 271 304 11036648 10.1016/s0076-6879(00)26060-6

[B22] Wei L Huhn JS Mory A Pathak HB Sosnovtsev SV Green KY Cameron CE Proteinase-polymerase precursor as the active form of ferine calicivirus RNA-dependent RNA polymerase J Virol 2001 75 1211 1219 10.1128/JVI.75.3.1211-1219.2001 11152494 PMC114027

[B23] Suzuki T Ito M Ezure T Kobayashi S Shikata M Tanimizu K Nishimura O Performance of expression vector, pTD1, in insect cell-free translation system J Biosci Bioeng 2006 102 69 71 10.1263/jbb.102.69 16952840

[B24] Zuker M Mfold web server for nucleic acid folding and hybridization prediction Nucleic Acids Res 2003 31 3406 3415 10.1093/nar/gkg595 12824337 PMC169194

[B25] Fullerton SWB Blaschke M Coutard B Gebhardt J Gorbalenya A Canard B Tucker PA Rohayem J Structural and functional characterization of sapovirus RNA-dependent RNA polymerase J Virol 2007 81 1858 1871 10.1128/JVI.01462-06 17121797 PMC1797576

[B26] Wellenreuther R Schupp I Poustka A Wiemann S The German cDNA Consortium SMART amplification combined cDNA size fractionation in order to obtain large full-length clones BMC Genomics 2004 5 36 10.1186/1471-2164-5-36 15198809 PMC436056

[B27] Tabuchi I Soramoto S Nemoto N Husimi Y An *in vitro* DNA virus for *in vitro* protein evolution FEBS Lett 2001 508 309 312 10.1016/S0014-5793(01)03075-7 11728441

[B28] Ezure T Suzuki T Higashide S Shintani E Endo K Kobayashi S Shikata M Ito M Tanimizu K Nishimura O Cell-free protein synthesis system prepared from insect cells by freeze-thawing Biotechnol Prog 2006 22 1570 1577 10.1021/bp060110v 17137303

[B29] Nishigaki K Taguchi K Kinoshita Y Aita T Husimi Y Y-ligation: an efficient method for ligating single-stranded DNAs and RNAs with T4 RNA ligase Mol Divers 1998 4 187 190 10.1023/A:1009644028931 10729904

