# Supplementary material for: Characterization of Norovirus RNA replicase for in vitro amplification of RNA
Source: BMC Biotechnol. 2013 Oct 9;13:85. doi: 10.1186/1472-6750-13-85 (PMC3852016; doi:10.1186/1472-6750-13-85)
Supplement: Additional file 1: Figure S1 — Amino acid sequence comparison of NV3Dpol proteins used in Rohayem’s report (A), in Fukushi’s report (B) and in this report (C). The Amino acid sequence homologies between (A) and (B), (B) and (C), (A) and (C) were 90.4, 99.2, 89.6%, respectively. Amino acid substitutions were indicated in bold letters. [file 1472-6750-13-85-S1.pdf]

Figure S1

(A) MGGDSKGTYCGAPILGPG**S**APKLSTKTKFWRSS**TT**PLPPGTYEPAYLGGKDPRVKGGPSLQQ
(B) MGGDDKGTYCGAPILGPG**N**APKLSTKTKFWRSS**NA**PLPPGTYEPAYLGGKDPRVKGGPSLQQ
(C) MGGDDKGTYCGAPILGPG**N**APKLSTKTKFWRSS**NA**PLPPGTYEPAYLGGKDPRVKGGPSLQQ

VMRDQLKPFTEPRGKPP**K**PSVLE**A**AKKTIINVLEQ**T**IDPP**E**KWS**FT**QACASLDKTTSSG**H**PHH**M**RK
VMRDQLKPFTEPRGKPP**N**PSVLE**S**AKKTIINVLEQ**V**IDPP**Q**KWS**YA**QACASLDKTTSSG**Y**PHH**V**RK
VMRDQLKPFTEPRGKPP**N**PSVLE**S**AKKTIINVLEQ**V**IDPP**Q**KWS**YA**QACASLDKTTSSG**Y**PHH**V**RK

ND**C**WNGESFTGKLADQASKANLM**F**EEGK**N**M**T**PVYT**G**ALKDELVKTDKIYGKIKK**R**LLWGSDL**A**TMI
ND**Y**WNGESFTGKLADQASKANLM**Y**EEGK**H**M**P**PVYT**A**ALKDELVKTDKIYGKIKK**R**LLWGSDL**S**TMI
ND**Y**WNGESFTGKLADQAPKANLM**Y**EEGK**H**M**P**PVYT**A**ALKDELVKTDKIYGKIKK**P**LLWGSDL**S**TMI

R**R**ARAFGGLMDELK**TH**C**V**TLP**I**RVGMNMNEDG**S**I**I**FE**R**HSR**HK**YHYDADYSRWDSTQQRAVLAAAL
R**C**ARAFGGLMDELK**AN**C**I**TLP**V**RVGMNMNEDG**P**I**M**FE**K**HSR**YR**YHYDADYSRWDSTQQRAVLAAAL
R**C**ARAFGGLMDELK**AN**C**I**TLP**V**RVGMNMNEDG**P**I**M**FE**K**HSR**YR**YHYDADYSRWDSTQQRAVLAAAL

VIMV**K**FS**S**EP**H**LAQ**V**VAEDLL**S**PSVVDVGDFKI**S**INEGLPSGVPCTSQWNSIAHWLLTLCALSEVT
EIMV**R**FS**A**EP**Q**LAQ**I**VAEDLL**A**PSVVDVGDFKI**T**INEGLPSGVPCTSQWNSIAHWLLTLCALSEVT
EIMV**R**FS**A**EP**Q**LAQ**I**VAEDLL**A**PSVVDVGDFKI**T**INEGLPSGVPCTSQWNSIAHWLLTLCALSEVT

NL**S**P**D**IIQANS**LF**SFYGDDEIVSTDIKLDPEKLTAK**L**K**K**YGLKPTRPDKTEGPLVISEDLNGLTFL
GL**G**P**D**IIQANS**MY**SFYGDDEIVSTDIKLDPEKLTAK**L**K**E**YGLKPTRPDKTEGPLVISEDLNGLTFL
GL**G**P**N**IIQANS**MY**SFYGDDEIVSTDIKLDPEKLTAK**F**K**E**YGLKPTRPDKTEGPLVISEDLNGLTFL

RRTVTRDPAGWFGKLEQSSILRQ**M**YWT**G**GPNHEDPSETMIPH**S**QRP**I**QLM**S**L**L**GE**AA**LHGP**A**FYSK
RRTVTRDPAGWFGKLEQSSILRQ**L**YWT**R**GPNHEDPSETMIPH**A**QRP**V**QLM**A**L**L**GE**SS**LHGP**S**FYSK
RRTVTRDPAGWFGKLEQSSILRQ**L**YWT**R**GPNHEDPSETMIPH**A**QRP**V**QLM**A**L**R**GE**SS**LHGP**S**FYSK

ISKLVI**A**ELKEGGMDFYVPRQE**P**MFRWMRFSDLSTWEGDRNLAPSFVNEDGVE
VSKLVI**S**ELKEGGMDFYVPRQE**S**MFRWMRFSDLSTWEGDRNLAPSFVNEDGVE
VSKLVI**S**ELKEGGMDFYVPRQE**S**MFRWMRFSDLSTWEGDRNLAPSFVNEDGVE

Amino acid sequence comparison of NV3D^{pol} proteins used in Rohayem's report (A), in Fukushi's report (B) and in this report (C). The Amino acid sequence homologies between (A) and (B), (B) and (C), (A) and (C) were 90.4, 99.2, 89.6 %, respectively. Amino acid substitutions were indicated in bold letters.
