# Supplementary material for: Characterization of Norovirus RNA replicase for in vitro amplification of RNA
Source: BMC Biotechnol. 2013 Oct 9;13:85. doi: 10.1186/1472-6750-13-85 (PMC3852016; doi:10.1186/1472-6750-13-85)
Supplement: Additional file 2: Figure S2 — Primer-independent RNA amplification reaction of Temp(GGG-CCC) RNA with NV3Dpol under the excess BSA condition (lane 3 and 4). Temp(GGG-CCC) RNA (5 pmol) was incubated with NV3Dpol (the amount was not detected) and sampled at 0 and 120 min. In the case of excess BSA +, 500 ng of BSA was added to the standard reaction mixture (BSA -). M1; 10 bp DNA step ladder (Promega). M2; DynaMarker dsRNA ladder (BioDynamics Laboratory Inc.). Ref.; Temp(GGG-CCC) RNA. [file 1472-6750-13-85-S2.pdf]

Figure S2

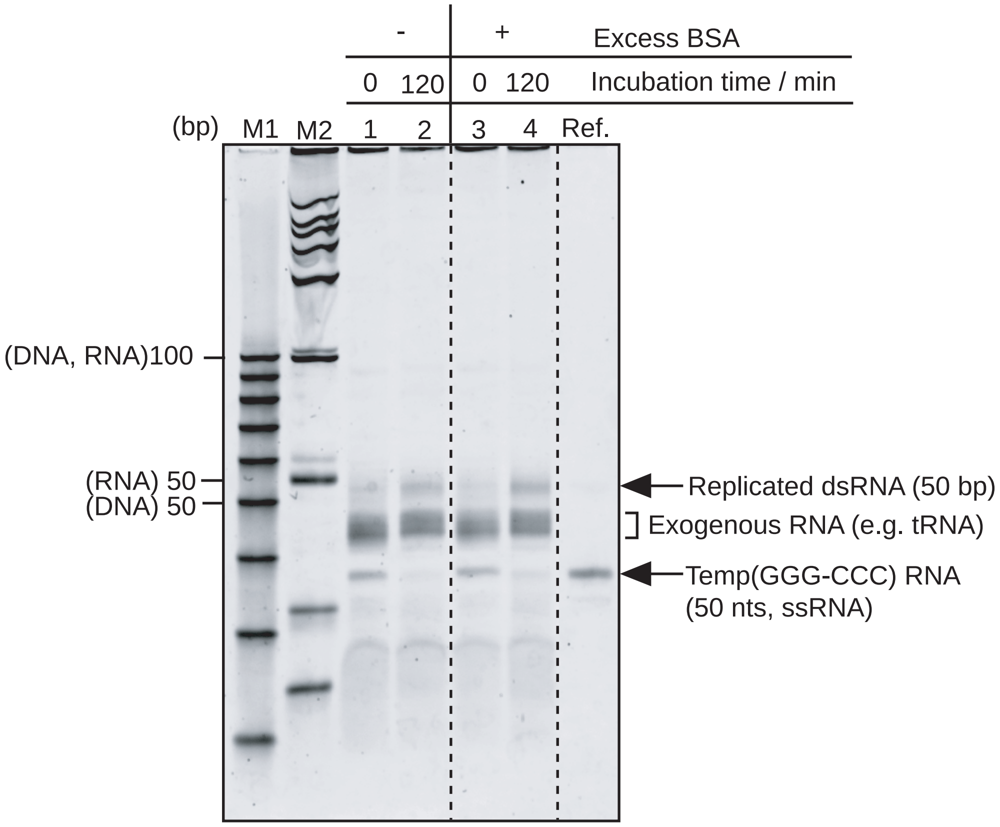

Primer-independent RNA amplification reaction of Temp(GGG-CCC) RNA with NV3D^{pol} under the excess BSA condition (lane 3 and 4). Temp(GGG-CCC) RNA (5 pmol) was incubated with NV3D^{pol}(the amount was not detected) and sampled at 0 and 120 min. In the case of excess BSA +, 500 ng of BSA was added to the standard reaction mixture (BSA -). M1; 10 bp step DNA ladder (Promega). M2; DynaMarker dsRNA ladder (BioDynamics Laboratory Inc.). Ref.; Temp(GGG-CCC) RNA
