# Supplementary material for: Characterization of Norovirus RNA replicase for in vitro amplification of RNA
Source: BMC Biotechnol. 2013 Oct 9;13:85. doi: 10.1186/1472-6750-13-85 (PMC3852016; doi:10.1186/1472-6750-13-85)
Supplement: Additional file 3: Figure S3 — Supplements to Figure 2. (A) Quantification of amplification of 50 nts ssRNA in Figure 2B (8M urea denaturing 10% PAGE). Closed circle; (i) Temp (GGG-CCC), closed diamond; (ii) Temp (GGG-GGG), closed triangle; (iii) Temp (GGG-CCA), closed square; (iv) Temp (GGG-UAC). (B) Non-denaturing PAGE. NV3Dpol (5 pmol) was incubated with (i) Temp (GGG-CCC), (ii) Temp (GGG-GGG), (iii) Temp (GGG-CCA) or (iv) Temp (GGG-UAC) (5 pmol each), and sampled at 0, 60, 120, 180 min respectively (reaction volume = 20 μL), which is the same reactions shown in Figure 2, Each reaction aliquots were analysed on a non-denaturing 10% PAGE, and imaged with Fx imager after SYBRgreenII staining. M; 10 bp DNA step ladder marker (Promega). Lot of the enzyme was different from the experiment of Figure 2. [file 1472-6750-13-85-S3.pdf]

Figure S3

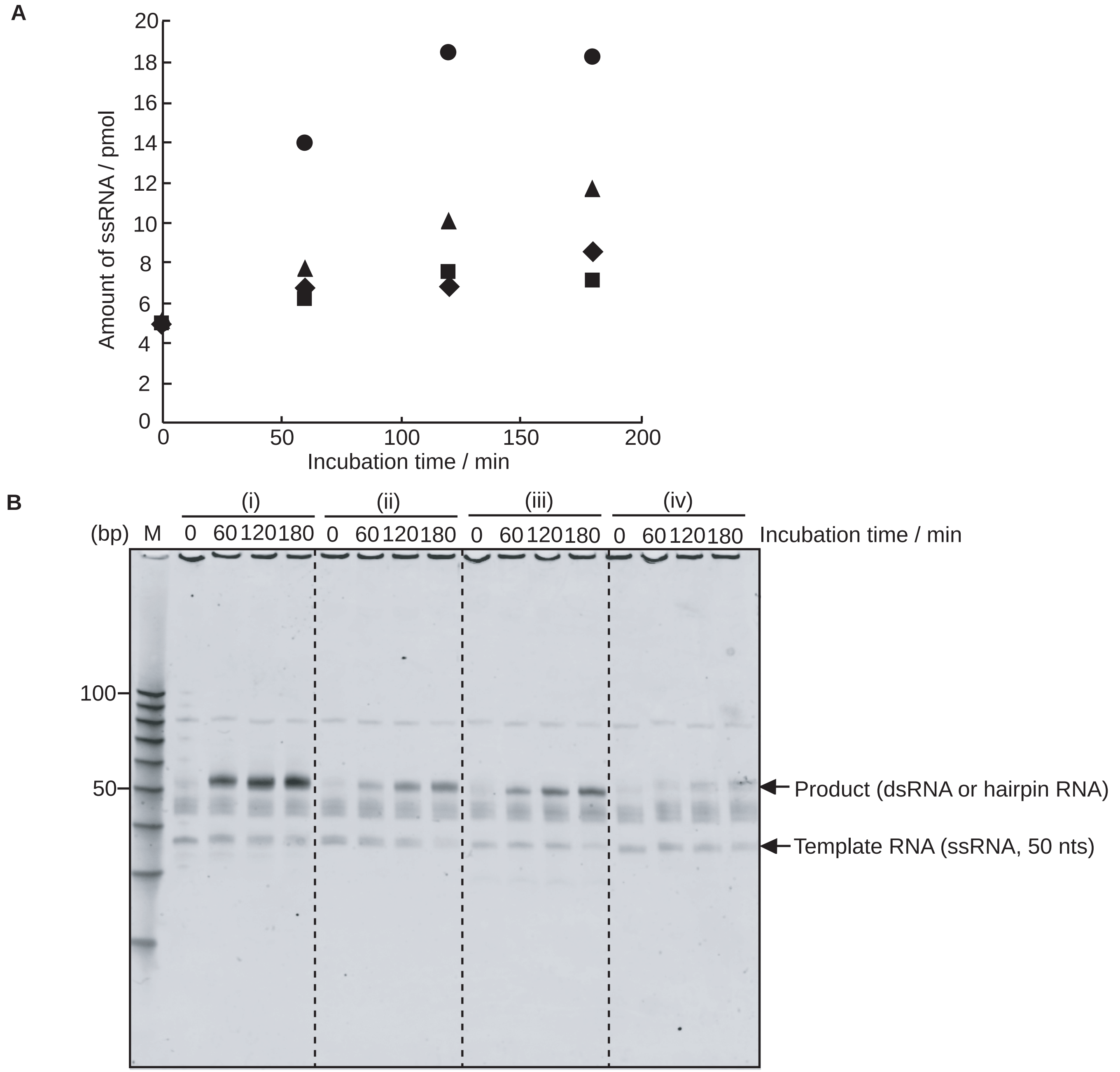

Supplements to Figure 2 (A). Quantification of amplification of 50 nts ssRNA in Figure 2B (8M Urea denaturing 10 % PAGE). Closed circle; (i) Temp(GGG-CCC), closed diamond; (ii) Temp(GGG-GGG), closed triangle; (iii) Temp(GGG-CCA), closed square; (iv) Temp(GGG-UAC). (B) Non-denaturing PAGE. NV3D^{pol}(5 pmol) was incubated with (i) Temp(GGG-CCC), (ii) Temp(GGG-GGG), (iii) Temp(GGG-CCA) or (iv) Temp(GGG-UAC) (5 pmol each), and sampled at 0, 60, 120, 180 min respectively (reaction volume = 20 micro L), which is the same reactions shown in figure 2. Each reaction aliquots were analyzed on non-denaturing 10 % PAGE, and imaged with Fx imager after SYBRgreenII staining. M; 10 bp DNA step ladder marker (Promega). Lot of the enzyme was different from the experiment of Figure 2.
