# Supplementary material for: Characterization of Norovirus RNA replicase for in vitro amplification of RNA
Source: BMC Biotechnol. 2013 Oct 9;13:85. doi: 10.1186/1472-6750-13-85 (PMC3852016; doi:10.1186/1472-6750-13-85)
Supplement: Additional file 4: Figure S4 — S1 nuclease treatment of the amplification product of Temp(GGG-CCC) . Temp(GGG-CCC) RNA (5 pmol) and NV3Dpol (the amount was not detected) were incubated at 30°C for 120 min. The reaction was stopped with adding EDTA, followed by PCI (phenol/chloroform/isoamylalchol = 25 : 24 : 1) and CIA (chloroform/isoamylalchol = 24 : 1) extraction, and ethanol precipitation. Then the pellet was resuspended with 1 × S1 nuclease buffer (TaKaRa) and incubated with 13.5 U/μL of S1 nuclease (TaKaRa) at 37°C for 15 min. Each aliquot was added with EDTA to stop the reaction, and analyzed on an 8M urea denaturing 10% PAGE (A) or a non-denaturing 10% PAGE (B), visualized by SYBRgreenII staining. M1; 10 bp step DNA ladder (Promega). M2; DynaMarker dsRNA ladder (BioDynamics Laboratory Inc.). White headarrows indicated in lane 2 were the nucleic acids from cell-free protein synthesis system (e.g. tRNAs). [file 1472-6750-13-85-S4.pdf]

Figure S4

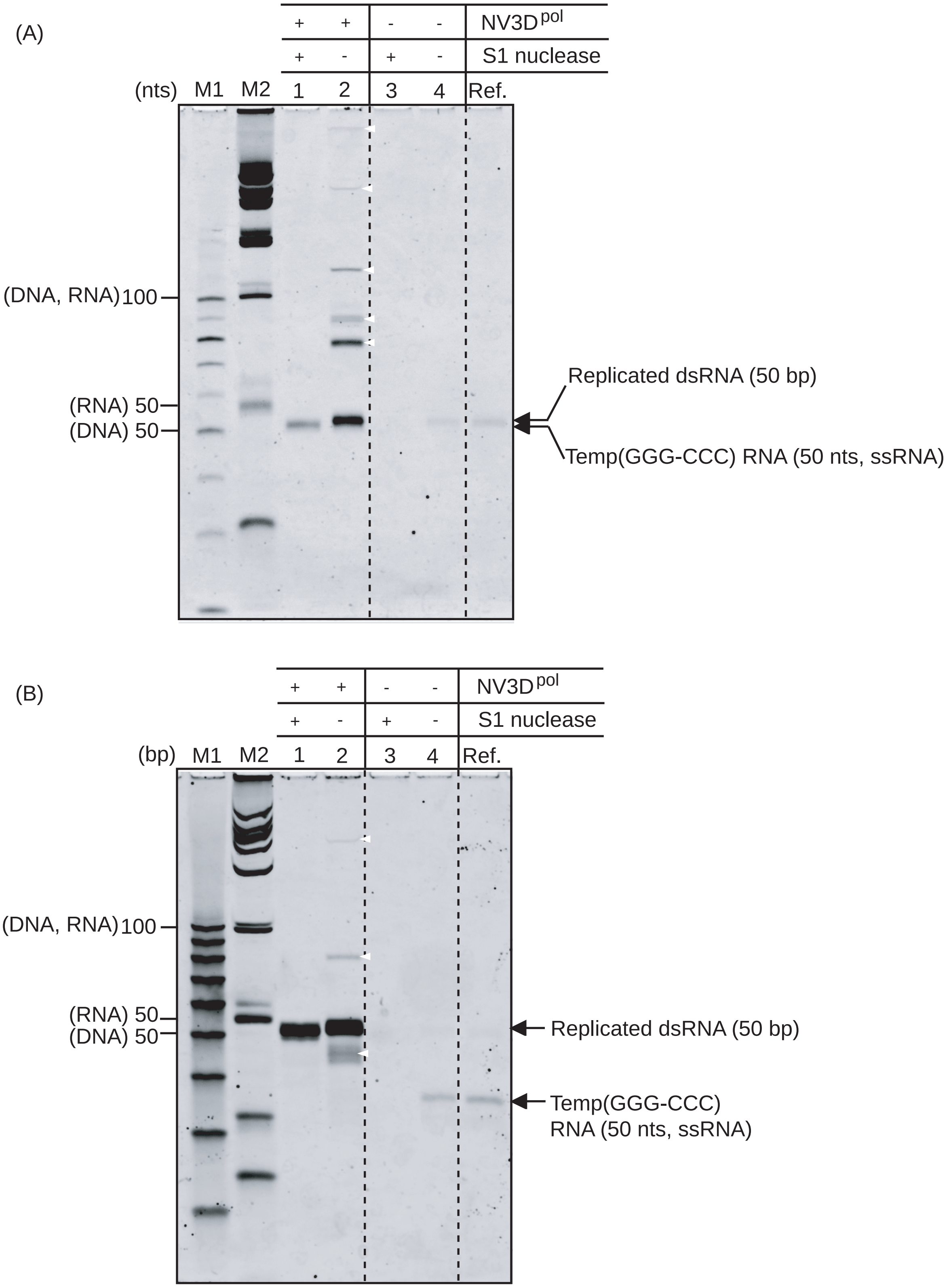

S1 nuclease treatment of the amplification product of Temp(GGG-CCC) RNA with NV3D^{pol}. Temp(GGG-CCC) RNA (5 pmol) and NV3D^{pol} (the amount was not detected) were incubated at 30°C for 120 minutes. The reaction was stopped with adding EDTA, followed by PCI (phenole/chloroform/isoamylalchol = 25 : 24 : 1) and CIA (chloroform/isoamylalchol = 24 : 1) extraction, and ethanol precipitation. Then the pellet was resuspended with 1 x S1 nuclease buffer (TaKaRa) and incubated with 13.5 U/micro L of S1 nuclease (TaKaRa) at 37°C for 15 minutes. Each aliquots were added with EDTA to stop the reaction, and analyzed on an 8M Urea denaturing 10 % PAGE (A) or a non-denaturing 10 % PAGE (B), visualized by SYBRgreenII staining. M1; 10 bp step DNA ladder (Promega). M2; DynaMarker dsRNA ladder (BioDynamics Laboratory Inc.). White headarrows indicated in lane 2 were the nucleic acids from cell-free protein synthesis system (e.g. tRNAs).
