# Supplementary material for: Characterization of Norovirus RNA replicase for in vitro amplification of RNA
Source: BMC Biotechnol. 2013 Oct 9;13:85. doi: 10.1186/1472-6750-13-85 (PMC3852016; doi:10.1186/1472-6750-13-85)
Supplement: Additional file 5: Figure S5 — Potential small stem-loop structures of 3’-terminus of RNA templates. (A) 3’-terminal sequence and potential small stem-loop structure of Temp(GGG-GGG) (ii), Temp(GGG-CCA) (iii) and Temp(GGG-UAC) (iv). Asterisks indicate the hybridization points between 3’-terminus. In (ii), the potential small stem-loop in the case of addition of 1 - 3 cytidine(s) on 3’-terminus were shown. (B) Potential small stem-loop structures of 3’-terminal sequence used in the previous reports ((a); [20,22], (b); [25]). [file 1472-6750-13-85-S5.pdf]

Figure S5

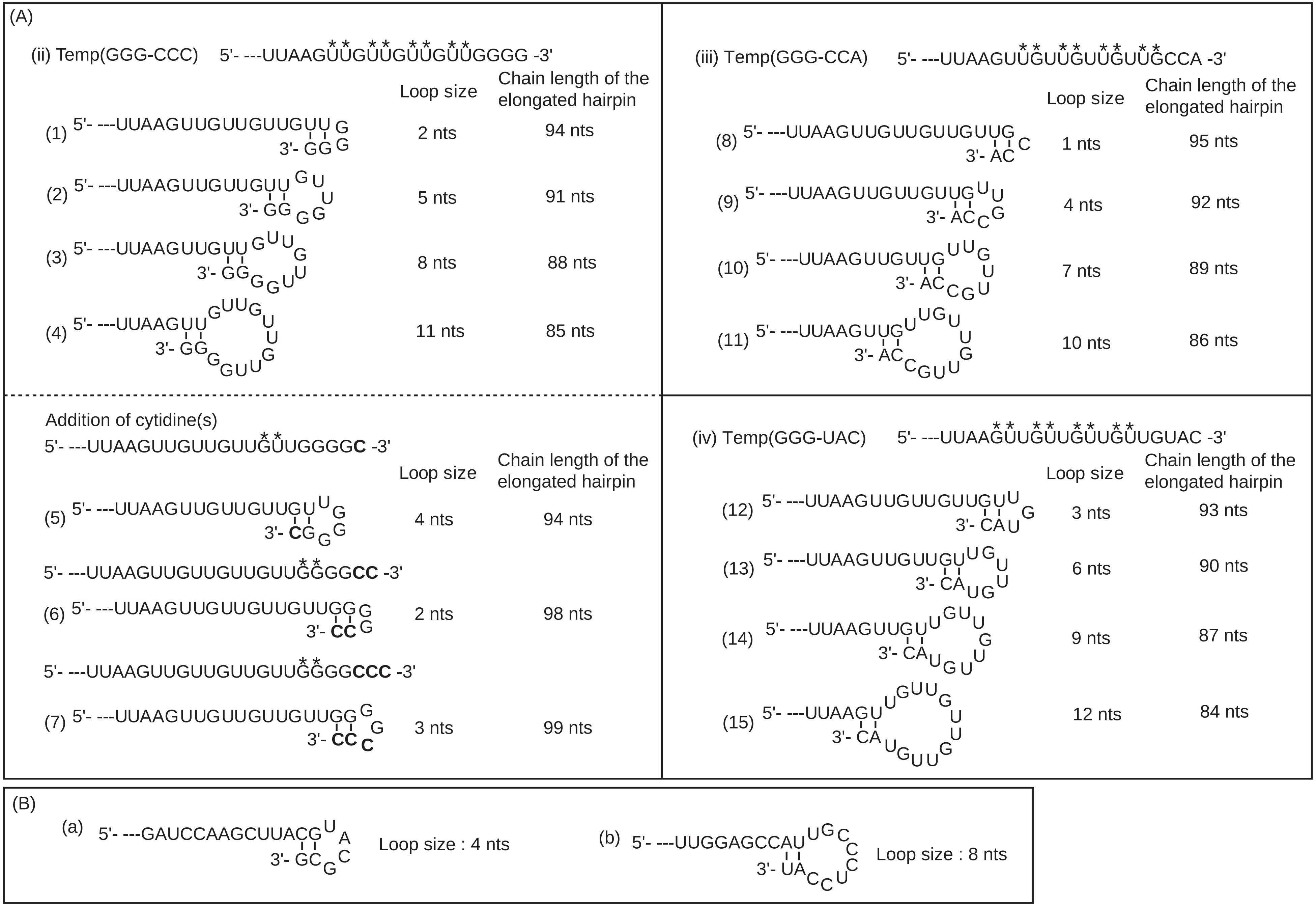

Potential small stem-loop structure of 3'-terminus of RNA templates. (A) 3'-terminal sequence and potential small stem-loop structure of Temp(GGG-GGG) (ii), Temp(GGG-CCA) (iii) and Temp(GGG-UAC) (iv). Asterisks indicate the hybridization points between 3'-terminus. In (ii), the potential small stem-loop in the case of addition of 1 - 3 cytidine(s) on 3'-terminus were shown. (B) Potential amall stem-loop structures of 3'-terminal sequence used in the previous reports ((a); [20, 22], (b); [25])
