# Supplementary material for: Characterization of Norovirus RNA replicase for in vitro amplification of RNA
Source: BMC Biotechnol. 2013 Oct 9;13:85. doi: 10.1186/1472-6750-13-85 (PMC3852016; doi:10.1186/1472-6750-13-85)
Supplement: Additional file 6: Figure S6 — Effect of poly(A)-tail for the initiation efficiency of RNA replication. TD257-735-A22 RNA (0.4 pmol) were incubated with NV3Dpol (4 pmol) (reaction volume = 20 μL) and analyzed on a non-denaturing 5% PAGE. M; 100 bp DNA ladder marker (Promega). In our PAGE condition, dsDNA 400 bp marker (Promega) corresponds to dsRNA 430bp marker (BioDynamics Laboratory Inc.). [file 1472-6750-13-85-S6.pdf]

Figure S6

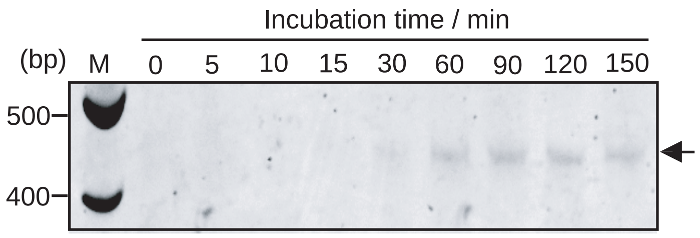

Effect of poly(A)-tail for the initiation efficiency of RNA replication. TD257-735-A_{22} RNA (4 pmol) were incubated with NV3D^{pol} (4 pmol) (reaction volume = 20 micro L) and analyzed on a non-denaturing 5% PAGE. M; 100 bp DNA ladder marker (Promega). In our PAGE condition, dsDNA 400 bp marker (Promega) corresponds to dsRNA 430bp marker (BioDynamics Laboratory Inc.).
