# Supplementary material for: Characterization of Norovirus RNA replicase for in vitro amplification of RNA
Source: BMC Biotechnol. 2013 Oct 9;13:85. doi: 10.1186/1472-6750-13-85 (PMC3852016; doi:10.1186/1472-6750-13-85)
Supplement: Additional file 7: Figure S7 — Supplement to Figure 8. Denaturing PAGE analysis. Experimental conditions are the same as in Figure 8, except the final analysis step, namely, analyzed on an 8M urea denaturing 10% PAGE, visualized by SYBRgreenII staining (A) or FITC fluorescence (B). Both images were merged (C). Arrowheads indicate the elongate primer (lane 1) and the primer-independent RNA synthesis product (lane 2), respectively. E, T, P and TP indicate NV3Dpol, RNA template, RNA primer and template-primer hybrid, respectively. +/- corresponds to the presence or absence of substrates. M; 10 bp DNA step ladder (Promega). [file 1472-6750-13-85-S7.pdf]

Figure S7

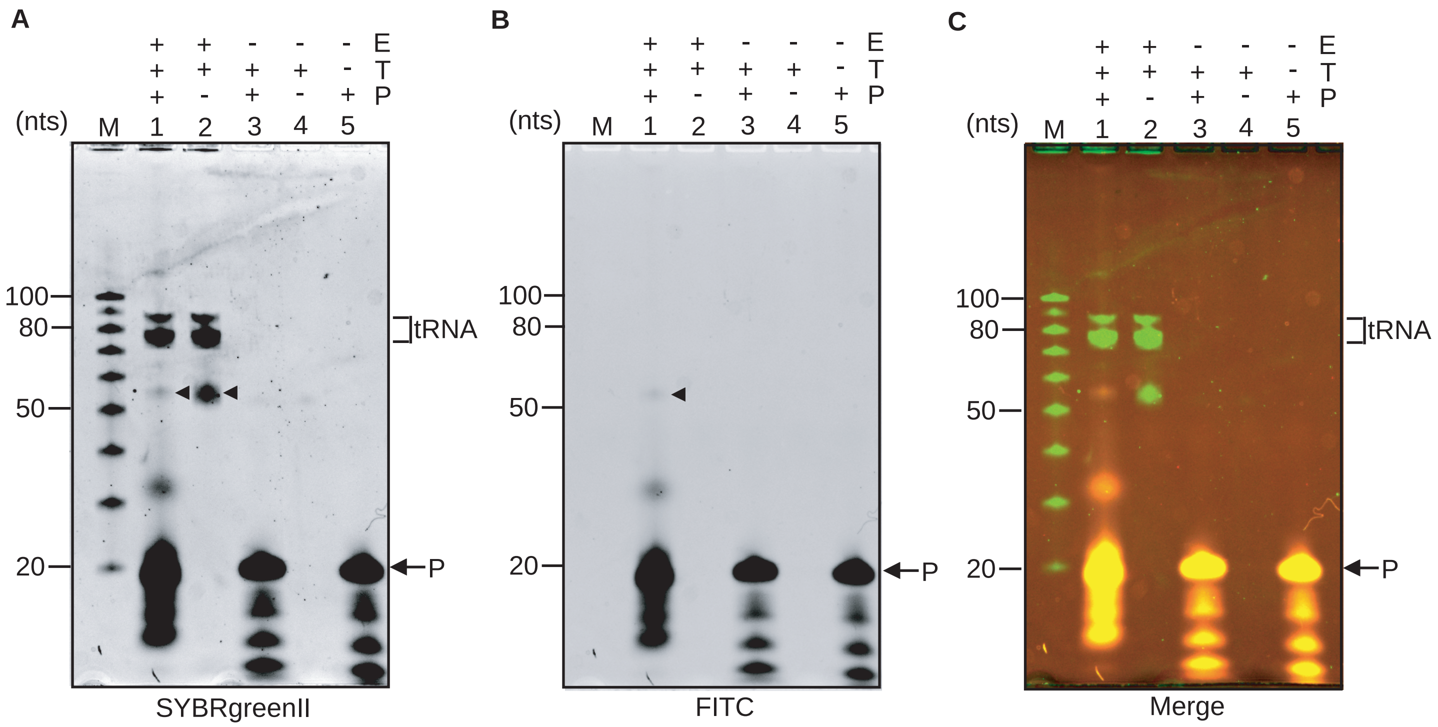

Supplement to Figure 8. Denaturing PAGE analysis. Experimental conditions are the same as in Figure 8, except the final analysis step, namely, analyzed on an 8M Urea denaturing 10 % PAGE, visualized by SYBRgreenII staining (A) or FITC fluorescence (B). Both images were merged (C). Arrowheads indicate the elongated primer (lane 1) and the primer-independent RNA synthesis product (lane 2), respectively. E, T, P and TP indicate NV3D^{pol}, RNA template, RNA primer and template-primer hybrid, respectively. +/- corresponds to the presence or absence of substrates. M; 10 bp DNA step ladder (Promega).
