# Supplementary material for: Characterization of Norovirus RNA replicase for in vitro amplification of RNA
Source: BMC Biotechnol. 2013 Oct 9;13:85. doi: 10.1186/1472-6750-13-85 (PMC3852016; doi:10.1186/1472-6750-13-85)
Supplement: Additional file 8: Table S1 — Sequences of RNA templates (a), DNA oligomers (b) used in this work. DNA sequence of 161-757 region in pTD1 (c). Underline indicates T7 ϕ 6.5 promoter sequence. [file 1472-6750-13-85-S8.pdf]

Table S1

Sequences of RNA templates (a), DNA oligomers (b) used in this work. DNA sequence of 161-757 region in pTD1 (c). Underline indicates T7 phi 6.5 promoter sequence.

	Name	Sequence (5' to 3')	Length (nts)
(a)	Temp(GGG-CCC)	GGGAAUAAGAUUUCACAGUUCAGAGAGACAUUAAGUUGUUGUUGUUGCCC	50
	Temp(GGG-GGG)	GGGAAUAAGAUUUCACAGUUCAGAGAGACAUUAAGUUGUUGUUGUUGGGG	50
	Temp(GGG-CCA)	GGGAAUAAGAUUUCACAGUUCAGAGAGACAUUAAGUUGUUGUUGUUGCCA	50
	Temp(GGG-UAC)	GGGAAUAAGAUUUCACAGUUCAGAGAGACAUUAAGUUGUUGUUGUUGUAC	50
	Temp(GGG-UUC)	GGGAAUAAGAUUUCACAGUUCAGAGAGACAUUAAGUUGUUGUUGUUGUUC	50
	Temp(GGG-UCC)	GGGAAUAAGAUUUCACAGUUCAGAGAGACAUUAAGUUGUUGUUGUUGUCC	50
	Temp(GGG-GCCCC)	GGGAAUAAGAUUUCACAGUUCAGAGAGACAUUAAGUUGUUGUUGUUGCCCC	51
	Temp(GGG-UCCCC)	GGGAAUAAGAUUUCACAGUUCAGAGAGACAUUAAGUUGUUGUUGUUCCCC	50
	Temp(GGG-UCCC)	GGGAAUAAGAUUUCACAGUUCAGAGAGACAUUAAGUUGUUGUUGUUCCC	49
(b)	Temp(GGG-CCC)	GCCAGTCGCCTGCAGTAATACGACTCACTATAGGGAATAAGATTTCACAGTTCAGAGAGACATTAAGTTGTTGTTGTTGCCC	82
	prT7tempGGG+	GCCAGTCGCCTGCAGTAATACGACTCACTA	30
	prTempCCC-	GGGCAACAACAACAACTTAATGTC	24
	prTempGGG-	CCCCAACAACAACAACTTAATGTCTC	26
	prTempCCA-	TGGCAACAACAACAACTTAATGTCTC	26
	prTempUAC-	GTACAACAACAACAACTTAATGTCTCTC	28
	prTempUUC-	GAACAACAACAACAACTTAATGTCTCTC	28
	prTempUCC-	GGACAACAACAACAACTTAATGTCTCTC	28
	prTempGCCCC-	GGGGCAACAACAACAACTTAATGTC	25
	prTempUCCCC-	GGGGAACAACAACAACTTAATGTCTCTC	28
	prTempUCCC-	GGGAACAACAACAACTTAATGTCTCTC	27
	prTD161-179	GCAGATTGTACTGAGAGTG	19
	prTD845-827	GGAAACAGCTATGACCATG	19
	prTD735-714	GCGGATAATATTTTGAACGACG	22
	prTD735-713g734c	GGGGATAATATTTTGAACGACGT	23
	prTD757-733	TTTTTTTTTTTTTTTTTTTTTTGCG	25
(c)	TD161-735	GCAGATTGTACTGAGAGTGCACCATATGCGGTGTGAAATACCGCACAGATGCGTAAGGAGAAAATACCGCATCAGGCCTTA<br>ATACGACTCACTATAGGAGTATTTTTATTCTTTCGTAAAAAAATTAGAAAAATAAAATATAAAGATATCGAATTCGAGCTCGGTA<br>CCCGGGGATCCTCTAGAGTCGGGCGGCTGTAAAACACGATACATTGTTATTAGTACATTTATTAAGCGCTAGATTCTGTGCG<br>TTGTTGATTTACAGACAATTGTTGTACGTATTTTAATAATTCATTAAATTTATAATCTTTAGGGTGGTATGTTAGAGCGAAAATC<br>AAATGATTTTCAGCGTCTTTATATCTGAATTTAAATATTAAATCCTCAATAGATTTGTAAAATAGGTTTCGATTAGTTTCAAACAA<br>GGGTTGTTTTTCCGAACCGATGGCTGGACTATCTAATGGATTTTCGCTCAACGCCACAAAACTTGCCAAATCTTGTAGCAG<br>CAATCTAGCTTTGTCGATATTCGTTTGTGTTTTGTTTTGTAATAAAGGTTCGACGTCGTTCAAAATATTATCCGC	575
