# Supplementary material for: Characterization of Norovirus RNA replicase for in vitro amplification of RNA
Source: BMC Biotechnol. 2013 Oct 9;13:85. doi: 10.1186/1472-6750-13-85 (PMC3852016; doi:10.1186/1472-6750-13-85)
Supplement: Additional file 9: Figure S8 — Schematic illustration of sample preparation for the 3’-terminus sequencing of RNA. As described in Materials and Methods, the reaction procedure is as follows; (1) the replicated RNA (RNA (-), shown in red) was ligated with an adapter DNA (shown in blue) hybridized at the 3’-terminal region of RNA(-) by the Y-ligation method. (2) The ligation product was reverse-transcribed with RT primer (green arrow) to cDNA, (3) and the cDNA was amplified by PCR, cloned and sequenced. [file 1472-6750-13-85-S9.pdf]

Figure S8

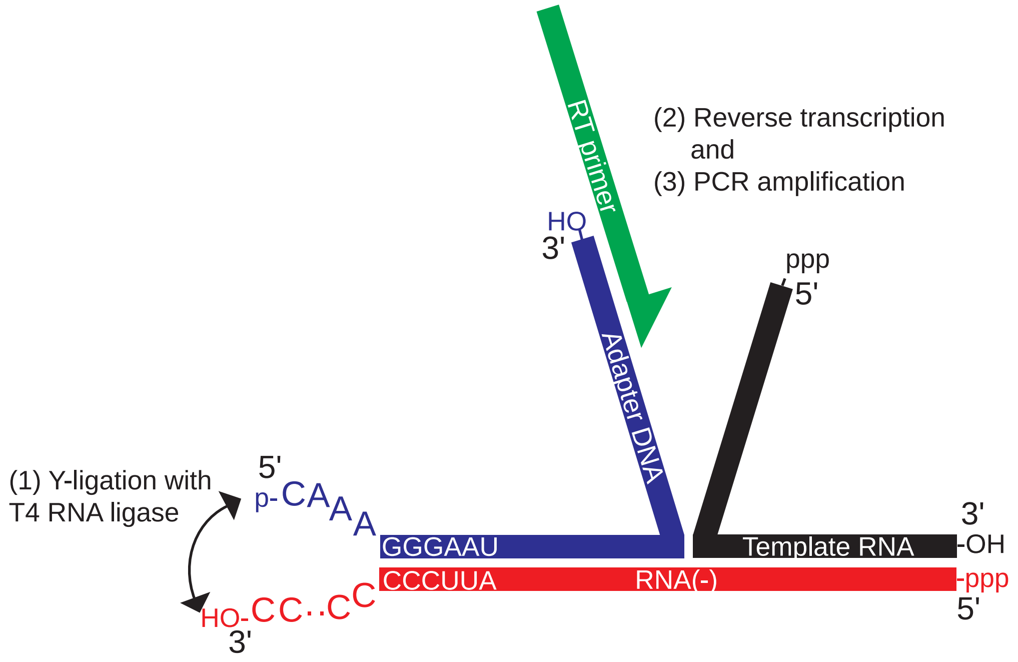

Schematic illustration of sample preparation for the 3'-terminus sequencing of RNA. As described in Materials and Methods, the reaction procedure is as follows; (1) the replicated RNA (RNA(-), shown in red) was ligated with an adapter DNA (shown in blue) hybridized at the 3'-terminal region of RNA(-) by the Y-ligation method. (2) The ligation product was reverse-transcribed with RT primer (green arrow) to cDNA, (3) and the cDNA was amplified by PCR, cloned and sequenced.
